# The Impact of HIV on Early Brain Aging—A Pathophysiological (Re)View

**DOI:** 10.3390/jcm13237031

**Published:** 2024-11-21

**Authors:** Mihai Lazar, Ruxandra Moroti, Ecaterina Constanta Barbu, Cristina Emilia Chitu-Tisu, Catalin Tiliscan, Teodora Maria Erculescu, Ruxandra Raluca Rosca, Stefan Frasila, Emma Teodora Schmilevschi, Vladimir Simion, George Theodor Duca, Isabela Felicia Padiu, Darie Ioan Andreescu, Andreea Nicoleta Anton, Cosmina Georgiana Pacurar, Patricia Maria Perdun, Alexandru Mihai Petre, Constantin Adrian Oprea, Adelina Maria Popescu, Enachiuc Maria, Daniela Adriana Ion, Mihaela Cristina Olariu

**Affiliations:** 1 Faculty of Medicine, University of Medicine and Pharmacy Carol Davila, No. 37, Dionisie Lupu Street, Sector 2, 020021 Bucharest, Romania; mihai.lazar@umfcd.ro (M.L.); ecaterina.barbu@umfcd.ro (E.C.B.); cristina.chitu@umfcd.ro (C.E.C.-T.); catalin.tiliscan@umfcd.ro (C.T.); teodora-maria.erculescu0720@stud.umfcd.ro (T.M.E.); ruxandra-raluca.rosca0720@stud.umfcd.ro (R.R.R.); stefan.frasila0721@stud.umfcd.ro (S.F.); emma-teodora.schmilevschi0720@stud.umfcd.ro (E.T.S.); vladimir.simion0720@stud.umfcd.ro (V.S.); george-theodor.duca0720@stud.umfcd.ro (G.T.D.); isabela-felicia.padiu0720@stud.umfcd.ro (I.F.P.); darie-ioan.andreescu0720@stud.umfcd.ro (D.I.A.); andreeanicoleta.anton0720@stud.umfcd.ro (A.N.A.); cosmina-georgiana.pacurar0720@stud.umfcd.ro (C.G.P.); patricia-maria.perdun0720@stud.umfcd.ro (P.M.P.); alexandru-mihai.petre0720@stud.umfcd.ro (A.M.P.); constantin-adrian.oprea0720@stud.umfcd.ro (C.A.O.); adelina-maria.popescu0720@stud.umfcd.ro (A.M.P.); maria.enachiuc0720@stud.umfcd.ro (E.M.); daniela.ion@umfcd.ro (D.A.I.); mihaela.olariu@umfcd.ro (M.C.O.); 2 National Institute for Infectious Diseases Prof. Dr. Matei Bals, No. 1, Calistrat Grozovici Street, Sector 2, 021105 Bucharest, Romania; 3 Faculty of Dental Medicine, University of Medicine and Pharmacy Carol Davila, No. 37, Dionisie Lupu Street, Sector 2, 020021 Bucharest, Romania

**Keywords:** HIV, brain, brain aging, neuroinflammation, HAART, HAND

## Abstract

**Background/Objectives**: This review aims to provide a comprehensive understanding of how HIV alters normal aging trajectories in the brain, presenting the HIV-related molecular mechanisms and pathophysiological pathways involved in brain aging. The review explores the roles of inflammation, oxidative stress, and viral persistence in the brain, highlighting how these factors contribute to neuronal damage and cognitive impairment and accelerate normal brain aging. Additionally, it also addresses the impact of antiretroviral therapy on brain aging and the biological markers associated with its occurrence. **Methods**: We extensively searched PubMed for English-language articles published from 2000 to 2024. The following keywords were used in the search: “HIV”, “brain”, “brain aging”, “neuroinflammation”, “HAART”, and “HAND”. This strategy yielded 250 articles for inclusion in our review. **Results:** A combination of blood-brain barrier dysfunction, with the direct effects of HIV on the central nervous system, chronic neuroinflammation, telomere shortening, neurogenesis impairments, and neurotoxicity associated with antiretroviral treatment (ART), alters and amplifies the mechanisms of normal brain aging. **Conclusions:** Current evidence suggests that HIV infection accelerates neurodegenerative processes of normal brain aging, leading to cognitive decline and structural brain changes at an earlier age than typically observed in the general population.

## 1. Introduction

Brain aging is a natural process characterized by gradual changes in both the structure and function of the brain as individuals grow older. These changes typically begin in early adulthood and progress slowly over decades. The most noticeable structural change is a reduction in brain volume, accompanied by a decrease in the number of synaptic connections, particularly in regions such as the frontal cortex and hippocampus, that are critical for cognitive functions including memory, decision-making, and emotional regulation [1,2].

Functionally, normal brain aging may lead to slower processing speeds, minor memory lapses, and difficulty with multitasking. At the cellular level, aging is associated with the accumulation of oxidative stress, reduced efficiency in cellular repair mechanisms, a gradual decline in neurotransmitter levels, mitochondrial dysfunction, and impaired lysosome and proteasome function [3,4,5,6,7]. Despite these changes, cognitive abilities such as vocabulary and general knowledge often remain stable or even improve with age, demonstrating the brain’s remarkable degree of plasticity. Thus, many older adults can maintain cognitive function and adapt to the challenges of aging through lifestyle factors such as physical activity, mental engagement, and social interaction.

Human immunodeficiency virus (HIV) has been a global health concern since its identification in the early 1980s. Over the past few decades, advances in antiretroviral therapy (ART) have significantly improved the life expectancy and quality of individuals with HIV, transforming what was once a fatal diagnosis into a manageable chronic condition [8,9]. However, as people living with HIV (PLWH) age, new challenges emerge, particularly concerning the long-term effects of HIV and its treatment on cognitive function and brain health [10].

Brain aging in PLWH has become a critical area of study, as it may contribute to increased susceptibility to neurocognitive disorders, including HIV-associated neurocognitive disorders (HAND) and possibly Alzheimer’s disease (AD). Understanding the interplay between HIV, aging, and cognitive decline is essential for developing effective interventions to preserve brain health in this population. The Frascati classification divides HAND into three categories, depending on the severity of the disease: asymptomatic neurocognitive impairment (ANI), mild neurocognitive disorder (MND), and HIV-associated dementia (HAD) [11]. Approximately 42.6% of people with HIV develop neurocognitive impairment, negatively affecting their quality of life, treatment adherence, employment, and overall mortality [12]. Although its etiology is not fully understood, there are multiple risk factors for brain aging, such as infections, advanced age, low CD4+ T cell nadir, a history of more severe immunosuppression, and possibly the use of ART [11]. The development of HAND is linked to synaptodendritic pruning and neuronal injury, caused by verotoxins (gp120, gp41, Tat, Vpr, Nef, and Rev), cytokines, and chemokines released by activated microglial cells, as well as increased extracellular glutamate, responsible for bioenergetic disturbances [11,13].

The mechanisms underlying brain aging in PLWH involve a combination of direct effects of the virus, chronic inflammation, neurotoxicity from ART, vasculopathy, and various comorbidities that compound the physiological age-related processes [14].

HIV disrupts the structure and function of the brain–blood barrier (BBB) by reducing pericyte coverage, altering cellular signaling and their interaction with endothelial cells [15]. A decrease in BBB integrity reduces the influx of vital nutrients into the brain and diminishes the blockage of pathogens, inflammatory cells, and other toxic agents, leading to early brain aging in PLWH [16,17]. HIV infection of microglia increases the production of neurotoxic mediators, accelerating the normal loss rate of myelination and the loss of white matter volume [18], while astrocyte infection contributes to the neuroinflammation process and the occurrence of HAND [19]. HIV infection is also associated with neurogenesis impairments and telomere shortening, further aggravating the process of early brain aging. ART may augment the structural and functional changes induced by the viral infection by inducing mitochondrial toxicity in neurons, microglia, and astrocytes [20,21,22], telomere shortening, cerebral subcortical atrophy, and demyelination [23,24,25,26]. ART is also associated with decreased N-acetylaspartate (NAA) levels in the frontal white matter (WM) [27] and promotes neuroinflammation and anemia, further altering the brain structure and functions.

However, there is currently a lack of understanding regarding the specific molecular mechanisms underlying the initiation and progression of brain aging in PLWH as well as the biological markers associated with this process. To address these gaps, this narrative review aims to highlight the impact of HIV on brain aging, presenting the HIV-related molecular mechanisms and pathophysiological pathways involved in brain aging, as well as the markers associated with its occurrence.

## 2. Materials and Methods

We extensively searched PubMed for English-language articles published from 2000 to 2024. The following keywords were used in the search: “HIV”, “brain”, “brain aging”, “neuroinflammation”, “HAND”, and “HAART”.

**Figure 1:**
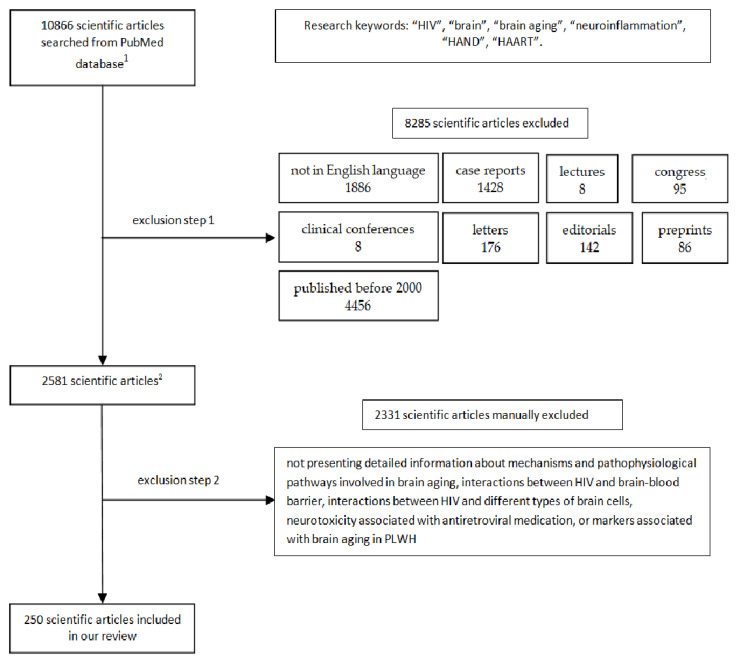
The flowchart of the review manuscript. ^1^ from 2020 until 2024; ^2^ if the information was unclear or contradictory, the review supervisors were consulted.

We excluded case reports, lectures, clinical conferences, letters, and editorials from the review. We selected articles that presented information regarding the mechanisms and pathophysiological pathways involved in brain aging, interactions between HIV and the brain–blood barrier (BBB), interactions between HIV and different types of brain cells, neurotoxicity associated with antiretroviral medication, and markers associated with brain aging in PLWH (Figure 1).

Although the brain’s involvement in HIV infection is well documented (5992 papers published after 2000 with the terms “HIV” and “brain” in the title or abstract, with 1276 remaining after the first exclusion step), when we searched for the terms “HIV” and “brain aging” in the title or abstract, we found only 41 papers (12 after the first exclusion step), with approximately 50% published in the last 5 years.

We initially searched the titles and abstracts to gather comprehensive information for our review. If the details were insufficient, we reviewed the full text of the articles. If the information was unclear or contradictory, the review supervisors were consulted.

Based on the strategy outlined above, we identified 250 articles to incorporate into our review (Figure 1).

Graphical representations were created using “www.biorender.com”.

## 3. Mechanisms Involved in Normal Brain Aging

One of the most significant changes associated with brain aging is cerebral atrophy [28]. The volume reduction of the cerebral parenchyma [29] and the widening of the ventricular system and sulci reflect this process in imaging studies [30,31]. However, age is not the only determinant of cerebral atrophy; hence, a distinction between normal and pathological brain aging may be essential for an early diagnosis of neurological diseases [28,32].

### 3.1. Cortical Atrophy

Gray matter volume decreases with age at a rate of 5.25% every 10 years [33]. However, its volume may also be influenced by modifiable lifestyle factors such as an unhealthy diet [34], smoking [35], or even physical activity [36]. Smoking negatively impacts the frontal lobe, primarily due to the long-term stimulation of cerebral nicotine receptors [37], while physical activity improves cerebral perfusion and enhances the synthesis of neurotrophic factors [38]. People Living with HIV (PLWH) experience accelerated musculoskeletal aging, particularly in the form of sarcopenia and osteoporosis, which leads to decreased physical activity [8,9,39]. Additionally, PLWH who smoke are more likely to experience greater neuronal damage, loss of myelin, brain atrophy, and poor cognitive performance [40].

Multiple studies explore the loss of gray matter in certain cerebral areas and the associated functional impairments. The most affected cortical regions in brain aging include those represented by the cingulate and somatomotor areas [41,42,43], with an overall decrease in gray matter volume being more pronounced in males than females [33]. Although there is no universal agreement regarding age-related changes in particular brain areas, memory loss, slower processing speed, and impaired motor activity are the most common dysfunctions associated with cerebral atrophy [42,43,44,45,46,47].

On a molecular level, oxidative stress increases with age [48] and correlates with gene alterations, impaired mitochondrial activity, and increased production of reactive oxygen species (ROS) [49]. ROS, produced at the mitochondrial level, induce the oxidation of lipids, proteins, and even DNA, further damaging the mitochondria (in vitro study) [50,51]. In the aging brain, slow metabolism affects the proper clearance of damaged products (in vitro study) [52]. Proteasome dysfunction further impairs protein degradation, leading to the accumulation of excessive products such as beta and tau amyloids [52,53]. Age-dependent changes in the blood-brain barrier (BBB) structures are also important factors that may lead to the degradation or shrinkage of human brain endothelial cells (HBEC), underexpression of tight junction (TJ) proteins, and morphological and metabolic transformations of pericytes and astrocytes contributing to brain aging (ex vivo studies) [54,55,56].

The HIV Tat protein increases the production of beta-amyloid, inhibits its degradation, and interacts with it, thereby promoting neurotoxicity. It also enhances the phosphorylation of the Tau protein, further contributing to neurotoxicity [57].

Although gray matter consists of neuronal bodies, dendritic ramifications, axons, and glial cells, age-related gray matter atrophy appears to be related to alterations of the neuronal architecture rather than the loss of neuronal cells [58,59]. On a cellular level, the aging process affects both neurons and glial cells, such as astrocytes and microglia [60]. Astrocytes undergo significant age-related changes, including shrinkage and mitochondrial dysfunction, which can impair their normal functions (preserving central nervous system (CNS) homeostasis, removing excessive glutamate, and contributing to BBB integrity) (ex vivo, in vitro studies) [54,61,62]. Microglial activation promotes inflammatory changes in brain tissue, increasing ROS production (ex vivo study) [63]. People Living with HIV (PLWH) experience accelerated “oxidative stress-induced aging”, which includes increased oxidation, mitochondrial dysfunction, early cell apoptosis, and ultimately, neurocognitive decline [23]. The HIV envelope glycoprotein gp120 amplifies oxidative stress and lipid peroxidation, leading to increased reactive oxygen species (ROS) and malondialdehyde levels, along with decreased intracellular glutathione, glutathione peroxidase, and superoxide dismutase activity [64], which induces apoptosis and cellular senescence (in vitro study) [65]. Additionally, the HIV Tat protein causes a significant decrease in telomerase activity [23,66], telomere length, and mitochondrial function, while increasing oxidative stress in human microglial cells [23]. Telomere structure becomes a target for oxidative damage, potentially acting as a key sensor of cell apoptosis induced by oxidative stress in PLWH [67].

### 3.2. Changes in White Matter

Age-related changes in white matter (WM) are most evident in the prefrontal cortex and the anterior corpus callosum, with progressive global involvement leading to deficient transmission of electrical signals across different brain regions [68,69,70,71].

WM aging is characterized by loss of myelination (demyelination), loss of volume (atrophy), tract disruption and axon degeneration, increased neuroinflammation, and vascular impairment [68,72,73,74,75,76].

#### 3.2.1. Oligodendrocyte Dysfunction

The loss of myelin and WM atrophy are primarily related to the oligodendrocyte dysfunction [68,77,78], induced by the following mechanisms:

(a) The decrease in cholesterol synthesis leads to reduced neuronal plasticity and myelin synthesis [79,80,81].
(b) The myelin-repairing function of aged oligodendrocytes is decreased because the activity of the enzyme TET1 (ten-eleven-translocation 1), which is essential for myelin repair in oligodendrocyte cells, decreases in aged individuals (in vitro study) [82,83].
(c) Increased iron levels associated with age can cause increased intracellular oxidation, resulting in oxidative damage to oligodendrocytes (in vitro study) [84,85,86].
(d) Oligodendrocytes are high metabolic demand cells, with intense oxidative reactions and low levels of glutathione, leading to a progressive age-related accumulation of oxidized lipids that may disturb the integrity of the myelin sheath (ex vivo, in vitro studies) [87,88,89,90,91].
(e) Degeneration of oligodendrocytes and oligodendrocyte progenitor cells (OPCs) is observed in the aging process of white matter. The decreased number and function of aged oligodendrocytes explain the age-related myelin degeneration and diminished renewal [77,78,92,93,94].
(f) Vascular impairment is closely linked to oligodendrocyte dysfunction due to the involvement of cerebral small vessel disease (CSVD) in aged oligodendrocyte dysfunction [95]. Age-related hypertensive vascular alterations can impair blood flow to the WM, leading to chronic ischemia (hypoperfusion), which further causes ischemia-induced apoptosis of oligodendrocytes and other glial cells [96,97]. Additionally, stiffening of the cerebral blood vessels that occurs with aging affects the pressure gradient in cerebral capillaries, causing extravascular fluid and solute accumulation in the interstitial space [98]. This free water excess in the brain interstitium may interfere with the integrity of WM, leading to axonal degeneration and demyelination over time [99].
(g) Aged oligodendrocyte dysfunction can be immune-mediated and associated with neuroinflammation [72]. Microglial cells, the resident immune cells within the WM, could drive age-related changes in oligodendrocytes [92,100,101,102].

The HIV Tat protein interacts with the N-methyl-D-aspartate receptor (NMDAR), leading to an increase in Ca^2^⁺ and CaMKIIβ levels. This interaction induces oligodendrocyte immaturity, reduces myelin-like membranes in mature oligodendrocytes, and can result in cell death [103].

#### 3.2.2. Neuroinflammation in the Aging Brain

Aging may cause low-grade chronic inflammation, also known as inflammaging [104,105].

The brain has a high energy expenditure and an extensive oxidative metabolism, resulting in increased production of reactive oxygen, nitrogen, and carbonyl species, collectively called RONCS. A high amount of RONCS is generated during aging and cannot be efficiently inactivated. These reactive species can interact with cellular components through non-enzymatic interactions such as glycation, leading to advanced glycation end products. These products further bind to pattern recognition receptors, known as receptors for advanced glycation endproducts (RAGE). RAGE stimulation activates the nuclear factor kappa-B (NF-kB), a transcription factor that increases pro-oxidative and proinflammatory responses. In turn, NF-kB increases RAGE expression, further activating NF-kB. This creates a vicious cycle that exacerbates neuroinflammation [104,106,107,108,109,110,111].

Neuroinflammation can be triggered by age-related DNA damage and cellular senescence. DNA damage stimulates various pro-inflammatory pathways, including DNA damage response (DDR)-associated activation of the NF-kB pathway and the cyclic GMP-AMP synthase (cGAS) stimulator of interferon genes (STING) pathway [92,112,113]. Cellular senescence leads to increased inflammatory activity of aged microglia, which displays a senescence-associated secretory phenotype (SASP) (ex vivo study) [114]. Moreover, interferon type 1 (IFN-1) signaling becomes elevated in aged microglia (ex vivo study) [92,115].

During normal brain aging, inflammatory activation of glial cells such as microglia and astrocytes occurs. These aged glial cells exhibit impaired physiological functions but enhanced inflammatory activity. The over-release of pro-inflammatory cytokines, increased production of nitric oxide and ROS, and elevated IFN-1 response are hallmarks of aged microglia [92,116,117,118,119].

(a) Microglial inflammatory activation

An abnormal age-related activation of microglia causes neuroinflammation [92,116,117]. The release of pro-inflammatory cytokines and free oxygen radicals by activated aged microglia may be directly toxic to oligodendrocyte lineage cells [68,120]. Inflammatory cytokines can also render the BBB leaky and dysfunctional, causing plasma protein build-up in the WM and accumulation of extracellular fluid (edema) in the interstitium, leading to WM lesions and loss of myelination [72,121,122].

Second, a specific form of less ramified microglia has been discovered in the WM of the aged brain, known as white-matter-associated microglia (WAM). Its function is to phagocytize (clear) myelin debris. In the aged brain, the WAM has decreased phagocytic function and an altered lipid metabolism, meaning these aged microglia can no longer efficiently clear myelin debris, resulting in the accumulation of lipid metabolites and damaged myelin, responsible for myelin sheath degeneration [82,123].

Furthermore, another type of microglia has been observed in the aged brain, referred to as lipid-droplet-accumulating microglia. They have several characteristic features, including impaired phagocytic function, increased reactive oxygen species, and the release of pro-inflammatory cytokines that are toxic to oligodendrocyte lineage cells (ex vivo studies) [124,125].

Finally, microglia conduct oligodendrocytes through immune modulation, manipulating the recruitment and differentiation of OPCs, thus facilitating the remyelination process. Aged microglia have impaired functions, leading to reduced myelination [92,100,101,102].

In PLWH, microglial inflammatory activation has an important role in neurogenesis impairment (in vitro study) [126]. HIV-microglia infection increases the production of neurotoxic mediators, accelerating the normal loss rate of myelination and loss of white matter volume [18].

(b) Astrocyte activation

Astrocytes release pro-myelination factors, providing lactate and cholesterol for myelin sheath synthesis [127,128]. Aged astrocytes have a proinflammatory phenotype, releasing inflammatory cytokines and ROS [105,106], which are toxic to oligodendrocyte lineage cells and enhance the recruitment and activation of peripheral immune cells in the CNS.

Astrocytes are regarded as reservoirs for HIV within the brain, which produce proteins as Tat with role in neuronal toxicity and synaptodendritic injury [129,130]. In PLWH, astrocyte infection increases the neuroinflammation process and occurrence of HAND [19].

### 3.3. Age-Related Bioenergetic Deficit

Age-related cerebral bioenergetic deficits are major drivers of cognitive impairment [131]. Diminished glucose availability and increased consumption are the main characteristics of the aging brain [131,132]. The decline in brain glucose uptake results from decreased expression of glucose transporters (GLUT), with glucose hypometabolism being especially pronounced in distal axons and their terminals [131,133]. Furthermore, age-dependent deterioration of respiratory enzymes results in mitochondrial dysfunction, particularly in neurons, which decreases glycolytic capacity, increases oxidative stress, and ultimately leads to neuronal apoptosis [131,134]. Astrocyte senescence is also associated with decreased aerobic glycolysis [131,135]. Another important feature is the decline in nicotinamide adenine dinucleotide (NAD) levels, which are significantly reduced in WM compared with gray matter [133]. The alteration of NAD redox homeostasis contributes to glucose hypometabolism, especially in gamma-aminobutyric acid (GABA)ergic neurons, impairing the function of sirtuins, such as SIRT3, leading to genomic instability and deterioration of neuronal availability [133,134]. The resulting lowered GABAergic tone is partly responsible for the hyperexcitability in normal aging brains; the outcome is increased energy demand through increased transmission, which further overwhelms the axonal bioenergetic capacity [133]. Brain hypoperfusion and dysfunction of the BBB may also be age-related, leading to diminished nutrients, further decreasing the substrate for adenosine triphosphate (ATP) production [70,136,137].

HIV infection is associated with altered glucose metabolism; increased GLUT1 expression by proinflammatory monocytes is a potential marker of inflammation in HIV-infected subjects [138].

PLWH also present increased oxidative stress, decreased oxidative phosphorylation, gluconeogenesis, ATP production and beta-oxidation, abnormal cell homeostasis, upregulation of mitochondrial DNA mutations, and cell apoptosis [139]. Progressive mitochondrial damage induced by HIV infection and antiretroviral treatment alters the mitochondrial metabolism, increasing the bio-energetic deficit, accelerating the aging process, senescence, and cell dysfunction [139].

### 3.4. Vascular Changes in the Aging Brain

Regarding vascular changes, the most common chronic disease associated with the aging brain is cerebral small vessel disease (CSVD). The condition affects the small arteries, arterioles, venules, and capillaries of the cerebral circulation [140] and is a primary contributor to a spectrum of cognitive disturbances known as vascular cognitive impairment [141,142]. The main symptoms associated with CSVD include stroke, cognitive decline, dementia, and other neuropsychological disorders [140].

Most authors agree on the existence of two main types of CSVD, both age-related: amyloidal and non-amyloidal [143]. The non-amyloidal type is the most common and is linked to arteriolosclerosis and hypertension and induces a progressive decrease in vascular lumen, with a reduction of cerebral blood flow and chronic cerebral hypoperfusion [140]. The result is bilateral areas of chronic ischemia, which can be visible on neuroimaging as reduced WM density on CT scans [140,144]. A more serious evolution of hypertensive small vessel disease may be associated with complete occlusion of the lumen, leading to small areas of acute ischemia, corresponding to lacunar infarction areas on neuroimaging [140].

Cerebral amyloid angiopathy (CAA) represents the second type of CSVD. CAA manifests through beta-amyloid accumulation in the walls of arteries and arterioles [140]. These changes are commonly observed in the aged population and can cause cognitive deterioration [145]. CAA also affects capillaries and can therefore alter BBB integrity; it is associated with microbleeds, microinfarcts, and hypoperfusion, and it has been considered an important factor contributing to Alzheimer’s disease [140,145].

HIV Tat protein accelerates the production of beta-amyloid and the phosphorylation of Tau protein, having complex interactions with their structures that promote the production of neurofibrillary tangles, a hallmark of Alzheimer’s disease [57].

Another important change in the aged brain is BBB dysfunction, leading to (a) decreased oligodendrocyte precursor cell maturation, impairing myelination and myelin repair [146]; (b) reduced expression of transporters such as GLUT1 and glycoprotein P (Pgp), leading to the impairment of both glucose influx and neurotoxic molecule efflux [147]; and (c) relocation of the aquaporin-4 molecules to the opposite side of the astrocyte end-foot, disrupting normal interstitial fluid flow [146]. These changes increase BBB permeability, followed by the extravasation of plasma components such as albumin and fibrinogen in the neural environment [140,147]. This contributes to neuroinflammation and impairs nutrient transport, resulting in further vasodilation and ultimately cognitive decline [146,147].

PLWH develop disruption of the structure and function of the BBB by reducing the pericyte coverage, altering cellular signaling, and their interaction with the endothelial cells [15]. Further, the loss of BBB integrity induces the reduction of vital nutrients entering the brain and the reduction of the blockage of pathogens, inflammatory cells, and other toxic agents, leading to early brain aging [16,17].

For a better overview, the main factors involved in normal brain aging and their pathophysiological pathways, as well as the impact of HIV on normal brain aging, are summarized in Table 1.

## 4. HIV-Related Molecular Mechanisms and Pathophysiological Pathways Involved in Brain Aging Acceleration

A combination of BBB dysfunction, with direct effects of HIV on the CNS, chronic neuroinflammation, and neurotoxicity from antiretroviral treatment (ART), alters and amplifies the mechanisms of normal brain aging, presented in Section 3.

### 4.1. HIV–BBB Interaction

The first interaction of HIV with the CNS occurs at the level of the BBB. The BBB structure (endothelial cells, pericytes, and astrocytes end-feets) has a vital role in allowing essential nutrients to enter the brain while blocking pathogens, inflammatory cells, and other toxic agents [16,17].

#### 4.1.1. The Pericyte

Pericytes are crucial for the maintenance and development of the BBB; they regulate cerebral blood flow through their contractile ability and influence the tightness of the BBB through paracrine signaling [149]. Although pericytes are found across different organs and vascular beds, with variations observed in the proportion of the endothelial abluminal surface they cover, the vasculature of the CNS has the highest pericyte distribution, featuring an endothelial cell-to-pericyte ratio of 1:1 to 3:1 and approximately 90% coverage of the abluminal surface [150]. Having a unique role as supporting cells, changes in the structure and function of pericytes caused by HIV infection lead to BBB breakdown [151]. A significant reduction in brain pericyte markers—CD13 and PDGFRβ (platelet-derived growth factor receptor beta)—has been documented in HIV-positive cases, both with and without ART, primarily linked to exposure to viral proteins (ex vivo study) [152].

Pro-inflammatory factors cause a 50–60% reduction in PDGFRβ expression in pericytes, and this loss leads to decreased pericyte coverage of HBEC and reduced angiopoietin-1 (Ang-1) production. Ang-1 is crucial for activating the TIE-2 receptor in endothelial cells and regulating endothelial stability. Consequently, the reduction of Ang-1 results in less pericyte coverage and instability of the BBB. Thus, as exemplified by the PDGFRβ-TIE-2 axis, HIV infection can disrupt the balance in the crucial interaction between pericytes and endothelial cells [15].

Additionally, PDGF-BB (platelet-derived growth factor BB), the ligand of PDGFRβ, has been shown to alter the pericyte secretome, leading to the increased production of growth factors and the pro-inflammatory cytokine IL-6, a major driver of neuroinflammation. This reduces the expression of TJ proteins, such as occludin and ZO-1, in endothelial cells, thereby decreasing BBB integrity (ex vivo, in vitro studies) [153,154]. HIV infection also modifies pericyte cellular signaling. Injury signals are transmitted from infected pericytes to neighboring cells via gap junctions and a multi-protein complex involving occludin, caveolin-1, and Alix. This multi-protein complex influences pericyte gene expression and membrane plasticity following infection [15].

The mechanisms by which HIV infects pericytes remain unclear. It has been confirmed that within 48 h of HIV infection, there is a pronounced increase in viral replication, accompanied by NF-κB acetylation and a reduction in occludin expression. These changes are associated with the activation of the SIRT-1 pathway, critical for regulating NF-κB acetylation. Although HIV’s exploitation of the SIRT-1 pathway for viral replication is well documented in other cell types, in pericytes, this process seems to occur through the targeted depletion of occludin. Notably, overexpression of occludin has been shown to reduce viral replication and suppress SIRT-1 activation. Multiple studies demonstrate that BBB pericytes can be productively infected by HIV both in vivo and in vitro [155].

In HIV entry, both the main HIV receptor CD4 and the co-receptors CCR5 and CXCR4 are typically used. Pericytes at the BBB express high levels of the HIV co-receptors CCR5 and CXCR4 while also expressing CD4 at lower levels. Due to the high expression of both CCR5 and CXCR4, pericytes can be infected by both CCR5- and CXCR4-tropic strains of HIV. The crucial role of these co-receptors in facilitating viral entry into BBB pericytes was highlighted by the effective inhibition of infection using maraviroc, a clinically approved CCR5 inhibitor, and AMD3100, a specific CXCR4 inhibitor [154]. Measurements of p24 levels and reverse transcriptase activity show that pericytes exhibit peak viral replication 2–3 days after HIV infection, followed by a steady decline after 7–10 days. This decrease in HIV production in BBB pericytes aligns with the increased viral genome integration into the host. Reactivation of latent HIV in these cells, marked by higher p24 and HIV RNA levels, can be triggered by histone deacetylase inhibitors and tumor necrosis factor (TNF). These findings highlight BBB pericytes as potential active and latent HIV reservoirs in the CNS (ex vivo study) [156].

Research has shown that HIV infection in pericytes correlates with increased blood-brain barrier (BBB) permeability and neuronal damage. HIV also significantly reduces BBB pericyte coverage in brain endothelium, further compromising barrier integrity and facilitating HIV entry into the CNS. As a result, aging-related increases in BBB permeability become excessively aggravated and persistent. Clinical manifestations that may be correlated to BBB dysfunction are various, including cognitive deficits, such as attention or memory deficits, altered executive function (decision-making and problem-solving or planning activity), and also motor impairments.

#### 4.1.2. The Impact of Viral Proteins on BBB (Trans-Activator of Transcription: Tat)

Tat protein has been shown to increase PDGF-BB expression in pericytes by activating the extracellular signal-regulated kinase (ERK) and c-Jun NH2-terminal protein kinase (JNK) mitogen-activated protein kinase (MAPK) pathways, further activating the NF-κB pathway. The increased PDGF-BB then triggers autocrine activation of the PDGFRβ signaling, promoting greater pericyte migration. While PDGF-BB is crucial for pericyte development, excessive levels lead to pericyte loss (in vitro study) [157].

It is well established that Tat plays a significant role in the development of HIV-associated neurocognitive disorders (HAND) [19]. It can alter gene regulation in the CNS and create a more pro-inflammatory brain environment, promoting HIV pathogenesis. Despite the presence of transcription factors like NF-κB and others that aid in transcription initiation from the integrated provirus, Tat is essential for enhancing polymerase processivity, increasing transcription initiation, and boosting viral mRNA production by over 100-fold, which is crucial for HIV replication [158].

Tat binds to various cell surface receptors and crosses the BBB without disrupting it, particularly affecting regions such as the occipital cortex, hypothalamus, and hippocampus (in vitro study) [159]. It may cross the BBB through adsorptive endocytosis, similar to HIV gp120, or via the mannose-6-phosphate receptor (M6PR) pathway (in vitro study) [160]. Tat exposure leads to the breakdown of tight junction (TJ) integrity, correlated with Ras homolog family member A (RhoA) activation and increased monocyte migration across the BBB (in vitro study) [161,162]. Activated monocytes exhibit an increased number of lysosomes, filopodia, and vesicular Golgi complexes, along with elevated levels of pro-inflammatory cytokines like IL-6, IL-10, and TNF-α. These changes further compromise the integrity of the BBB (in vitro study) [163]. Tat can weaken the TJs of brain endothelial cells by affecting occludin. It inhibits occludin mRNA expression via the RhoA/ROCK signaling pathway—a process partly mitigated by inhibitors of p160-Rho-associated coiled-coil kinase (ROCK) and RhoA inhibitor C3 exoenzyme. Additionally, it promotes occludin cleavage through matrix metalloproteinase 9 (MMP-9) (in vitro study) [164]. Tat can also trigger nuclear localization of ZO-1 via the Rho signaling pathway, which appears to be a cAMP response element-binding protein-dependent response. In sum, total ZO-1 levels drop while ZO-1 expression in the nucleus is upregulated [165].

Scientific evidence supports the role of the Tat protein as a mediator of neurotoxicity, primarily through excitotoxicity. This process involves increased cytosolic calcium, mitochondrial calcium uptake, ROS generation, caspase activation, and ultimately, neuronal death. Elevated ROS production, which damages mitochondrial function, is also observed in normal brain aging. Thus, oxidative stress induced by Tat may serve as a virus-specific mechanism accelerating brain aging in HIV-infected individuals.

#### 4.1.3. The Impact of Viral Proteins on BBB (Envelope Glycoprotein 120: gp120)

Gp120 is an HIV envelope glycoprotein involved in viral attachment and entry into cells. It initially mediates attachment by binding to the CD4 receptor, triggering a conformational change that allows membrane fusion between the virus and host cell mediated by the CCR5 coreceptor [166].

Despite the lack of CD4 receptors expressed on BBB endothelium, gp120 can mediate HIV entry into endothelial cells through adsorptive endocytosis in a lectin-like manner, providing a means for the passage of the virus and its envelope protein across the BBB without affecting its integrity (animal model study) [167].

Moreover, the passage of the virus through the endothelial cells of the BBB does not necessarily involve a productive infection, as observed in vitro on brain microvascular endothelial cells (ex vivo study) [168].

The toxicity gp120 exhibits on brain endothelial cells is well established (ex vivo study) [169]. Gp120 induces programmed cell death of endothelial cells in both in vitro and in vivo studies, possibly through a caspase-3-mediated mechanism (in vitro study) [170,171]. On human brain microvascular endothelial cells (HBEC), gp120 exhibited cytotoxic effects in conjunction with IFN-γ via a MAPK cellular pathway, but only on cells derived from children, not adults (in vitro study) [172].

Moreover, gp120 increases oxidative stress and lipid peroxidation, with increased ROS and malondialdehyde levels and decreased intracellular glutathione, glutathione peroxidase, and superoxide dismutase activity [64]. An increase in oxidative stress was also observed in human aortic endothelial cells alongside a higher proneness to apoptosis and cellular senescence, following a rise in microvesicle production induced by CXCR4 gp120 (in vitro study) [65].

Activation of metalloproteinases mediated by gp120 may play an important role in BBB disruption, as seen in vitro in the caudate putamen of rats, by inducing a decrease in laminin, altering the structure of the basement membrane of the BBB, resulting in a decrease in claudin-5 and occludin, which affects TJ structure [172].

In vitro studies on human BBB models revealed that HBMEC, used as a substitute for endothelial cells of the BBB, expresses CXCR4 and CCR5, co-receptors involved in HIV infection of target cells, and gp120 can trigger a reduction in an array of TJ proteins (ZO-1, ZO-2, and occludin) expressed by an HBMEC monolayer (in vitro study) [173]. Gp120 can interact with endothelial cells through these co-receptors, inducing an elevation in Ca^2+^ flux and protein kinase C (PKC) activation, which results in decreased BBB tightness and increased monocytic passage across it (in vitro study) [174].

Therefore, evidence shows that HIV-1 gp120 protein is implicated in MMP activation, increasing the release of MMP-2 and MMP-9 in neurons. Their characteristic activity is gelatinolytic, affecting neurons, the vessel matrix, and endothelial cells (as they are gelatinases). Consequently, a weakened BBB with serum protein leakage has been observed more frequently in HIV-infected patients with dementia than in those with no cognitive deficit.

Moreover, gp120 induces increased secretion of IL-6 and IL-8 in HBEC due to STAT pathway activation, which consequently promotes monocytic adhesion and migration across this BBB model (ex vivo study) [175].

#### 4.1.4. The Impact of Viral Proteins on BBB (Negative Regulatory Factor: Nef)

Nef is a 27–35 kDa HIV/SIV protein that enhances viral infectivity and supports viral replication in mononuclear cells in peripheral blood [176].

In vitro BBB models exhibited increased permeability after interaction with Nef-exosomes released from HIV-infected microglia, which also decreased ZO-1 expression in HBEC (in vitro study) [177].

Primary human fetal astrocytes alongside SVGA cells (an immortalized astrocytic cell line) exposed to Nef protein produced greater amounts of IL-6 and IL-8, which have been linked to increased endothelial layer permeability (in vitro study) [178,179].

Moreover, the Nef HIV-associated protein may help the virus evade the immune response by inducing reduced expression of both MHC II and MHC I and increased expression of immature MHC II complexes, which may further disrupt the BBB (in vitro study) [180].

Nef protein is also able to influence the transcription process of MMPs at the BBB, resulting in the disruption of the BBB. Nef expression has been correlated with AIDS-associated dementia through evidence from brain tissue samples derived from patients with AIDS who died with moderate to severe dementia [181]. 

#### 4.1.5. Monocytes Transmigration

An important phenomenon contributing to HIV infiltration into the CNS is the transmigration of CD14+CD16+-infected mature monocytes across the BBB (in vitro study) [182]. There are two types of transmigration: paracellular and transcellular. The paracellular route predominates in the acute phase, while the transcellular route is more prevalent in the chronic phase (in vitro study) [183].

The acute phase occurs between 4 and 8 days after peripheral infection, and it contributes significantly to the seeding of HIV reservoirs in the CNS (in vitro study) [184]. It has been observed that CCL2 chemokine and junctional proteins JAM-A (junctional adhesion molecule A) and ALCAM (activated leukocyte cell adhesion molecule), expressed on the surface of both monocytes and endothelial cells, play a key role in the transmigration of monocytes across the BBB during the acute phase of infection [184,185]. The increased expression of JAM-A and ALCAM on the monocytes in HIV infection is induced directly by the infection of monocytes and is not secondary to viral proteins’ exposure or by cytokines associated with HIV infection [184]. In the monocyte transmigration process, ALCAM expressed by the endothelial cells of the BBB binds to ALCAM on the monocytes, whereas JAM-A of the endothelial cells binds to lymphocyte function-associated antigen 1 (LFA-1) on the monocytes [185]. The CCL2 protein expressed on the apical surface of brain microvascular endothelial cells of the BBB binds to CCR2 on the surface of monocytes, triggering a signaling pathway that leads to the activation of LFA-1 and very late antigen-4 (in vitro study) [186]. These monocyte molecules bind to their endothelial targets (ICAM-1 and VCAM-1), increasing the transmigration of infected monocytes into the CNS. The transmigration of infected monocytes through the BBB occurs even with suppressive ART; therefore, blocking CCR2, JAM-A, and ALCAM presents potential therapeutic strategies to reduce or even completely stop HIV-infected transmigration across the BBB [184]. Treatments with cenicriviroc, JAM-A antibodies, and ALCAM antibodies have been shown to significantly reduce the entry and reseeding of the CNS [182].

Dopamine binds to the D1 receptor of partially transmigrated monocytes through the inter-endothelial junctions of the BBB, inducing changes in tubulin and actin localized to membrane protrusions, thereby accelerating the process of monocyte transmigration [187]. Dopamine also activates the metalloproteinase ADAM 17 in monocytes, facilitating their diapedesis across epithelial junctions (in vitro study) [186]. The type of monocytes affected by these processes is CD14+CD16+, which are found in the peripheral blood of HIV-infected individuals compared with healthy individuals, who predominantly exhibit a CD14+CD16− profile [186,187].

The patients who develop HIV-associated dementia exhibit elevated and persistent extracellular ATP levels in the serum (eATP) from the early onset of symptoms, even though most of them have HIV undetectable viral loads in the blood on ART [183]. These elevated levels, frequently found in the chronic phase of HIV infection, promote selective transcellular transmigration of infected monocytes through the BBB [183]. This type of transmigration elicited by eATP does not involve CCL2 mediation, as chronic exposure to elevated levels of eATP does not increase CCL2 expression compared with basal conditions [183]. The transmigration process of infected monocytes in response to elevated eATP levels involves key components such as JAM-A (expressed on both monocyte and endothelial surface cells) and LFA-1 proteins (expressed only on monocyte surfaces, not on endothelial cells) [183,185].

Monocyte transmigration into the CNS is a major mechanism responsible for the initiation and propagation of the HIV infection into the brain parenchyma, establishing the CNS as a viral reservoir. The resulting neuroinflammation promoted by monocyte/macrophage activation persists in the CNS despite ART administration, significantly contributing to the pathogenesis of HAND.

Viral DNA from circulating CD14^+^CD16^+^ monocytes was found to be associated with cognitive impairment, such as deficits in motor skills and motor speed, and also altered verbal memory and executive function [188].

### 4.2. HIV Interaction with CNS Cells

The presence of HIV in the CNS can be detected within two weeks of primary infection (in vitro study) [189]. The transmigration of infected CD4+ T cells and macrophages across a highly permeable BBB, especially in the initial stages of infection, can lead to the infection of CNS cells: CD4+ T cells and macrophages can persist in the CNS or propagate the infection to CNS cells (ex vivo study) [190]. Studies using hybridization techniques and immunohistochemistry have identified the presence of HIV-DNA in astrocytes, microglia, and macrophages, suggesting these cells as potential targets for improved suppressive ART (ex vivo, in vitro studies) [129,191,192]. The persistence of proviruses at the CNS level under ART has been demonstrated in the basal ganglia, frontal lobe, thalamus, and periventricular WM [190].

#### 4.2.1. HIV Interaction with Microglia

Microglia do not express CD4 receptors, but they do express CCR5 and CXCR4 co-receptors on their surface. HIV uses its envelope glycoprotein gp120 to bind to these co-receptors to enter the microglia (in vitro study) [193]. The interaction between gp120 and CCR5 or CXCR4 facilitates the fusion of the viral membrane with the host cell membrane, allowing the viral RNA to enter the microglia (in vitro study) [193]. Moreover, gp120 induces TNF-α-dependent apoptosis and the production of IL-1 and IL-1β in microglia [18]. IL-1β signaling is crucial for both initiating and sustaining neuroinflammation, leading to the production of neurotoxic mediators, including TNF-α, nitric oxide (NO), and cyclooxygenase-2, and increased intracellular calcium ion load and ROS [18], thereby accelerating the normal loss rate of myelination and WM atrophy. It can be stated that microglia, which can be triggered by an extensive list of pro-inflammatory stimuli, including HIV proteins, represent a continuous source of numerous neurotoxic molecules, as well as a site of viral replication. The microglial pro-inflammatory response (with cytokines and reactive oxygen species) enhances neuron damage (reactive microgliosis), resulting in the loss of neurons and progression of HAND. The disrupted microglial function translates into clinical signs of aging in HIV patients, such as confusion, distal symmetric polyneuropathy, and paraesthesias.

#### 4.2.2. HIV Interaction with Astrocytes

Astrocyte infection mainly occurs through cell-to-cell contact and endocytosis, but only a small fraction of cells become infected, mostly in a non-productive manner [129,191]. Given their high prevalence in the brain and long lifespan, astrocytes are regarded as reservoirs for HIV within the brain [129]. Although HIV does not productively infect astrocytes, they have been shown to produce non-structural proteins such as Tat (in vitro study) [194]. The Tat protein is a crucial virulence factor of HIV, essential for the virus’s replication and transmission [195]. In astrocytes, gap junction communication is inhibited during inflammatory conditions to control infection [196]. However, in HIV infection, the Tat protein maintains functional gap junctional communication by upregulating the expression of a crucial gap junction component, connexin43 (Cx43) [196]. Thus, the transfer of Tat to uninfected cells is sustained [196], promoting HIV pathogenesis by altering gene regulation, increasing the neuroinflammation process, and contributing to the occurrence of HAND [19]. Considering these data, it is likely that astrocytes shift to a reactive state and have important contributions to HAND pathology through indirect mechanisms that imply disruption in the astrocyte glutamate transporters followed by glutamate imbalance in the brain and consequently excitotoxicity of neurons. HIV patients may develop neuropsychiatric manifestations such as mood alterations, insomnia, and dizziness.

#### 4.2.3. HIV’s Impact on Neurons

HIV cannot directly infect neurons; thus, neuronal damage and death in HAND must be mediated through indirect mechanisms (in vitro study) [197]. The Tat protein can induce apoptosis in human neurons, by binding to the low-density lipoprotein receptor-related protein (LRP) in these neurons [197]. This binding leads to a macromolecular complex at the neuronal cell membrane involving LRP and NMDAR, facilitated by the scaffolding protein PSD-95 [197]. Tat induces tyrosine phosphorylation of the NMDAR subunit 2A [197]. NMDAR phosphorylation leads to changes in NMDAR activity, modifications in protein-protein interactions, and alterations in mitochondrial trafficking, thus impairing cognitive functions [197]. HIV’s viral gp120 can also contribute to neuronal damage through pathways that involve disrupted calcium balance, induction of the pro-apoptotic transcription factor, activation of oxidative stress, and alterations in mitochondrial fission and fusion by dysregulating mitochondrial function in neurons (in vitro study) [198]. Along with Tat, gp120 triggers the perinuclear accumulation and aggregation of damaged mitochondria, followed by mitochondrial fission driven by the translocation of dynamin-related protein 1 (DRP1) to these compromised mitochondria [199]. Additionally, mitochondrial fragmentation begins, marked by the recruitment of PINK1 (PTEN-induced putative kinase 1) to the damaged mitochondria, leading to the translocation of Parkin, the recruitment of SQSTM1, and further mitochondrial aggregation [199]. Although mitophagy is initiated, the buildup of SQSTM1 suggests that the process is incomplete. Moreover, gp120 and Tat impair mitochondrial function by reducing mitochondrial membrane potential, which is linked to DRP1-mediated fission [199].

These mechanisms explain that impairment in mitochondrial function and their distribution are significant mechanisms of HIV neuronal damage. The altered neuronal mitochondria are characterized by impaired respiration (also consecutive to HIV viral gp120 presence) that leads to bioenergetic dysfunction and production of ROS. The increased production of ROS is frequently associated with neuronal dysfunction and aging; thereby, it can be stated that HIV-infected patients may develop a pattern of brain degradation similar to the aging phenomenon. 

Mitochondrial disturbances in neurons translate into clinical manifestations such as cognitive disorders: poor performance in verbal fluency, information processing speed, working memory, and executive functioning, and also mood alterations, sleep disorders (insomnia, disturbing dreams), headaches, and dizziness.

#### 4.2.4. HIV-Macrophage Interaction

CD16+ macrophages are more susceptible to HIV infection than other populations and are found in greater numbers in the peripheral blood of HIV-infected patients. Diapedesis across the BBB is facilitated by junctional proteins such as JAM-A, ALCAM, PECAM-1, and CD99, which are preferentially expressed on the surface of CD16+ macrophages. Based on the expression of CD14, two subpopulations of CD16+ macrophages can be defined. The CD16+CD14+ type shows improved transmigration capacity across the BBB compared with the low CD14 type, consecutive to their increased JAM-A and ALCAM expression [189].

Monocyte activation and migration contribute to persistent neuroinflammation, proven to be a strong mediator of neuronal deterioration in HAND.

The influence of the underlying inflammatory process remains controversial, as different studies report modest changes in receptor transcript expression without influencing infectivity or downregulating CD4 (ex vivo, in vitro studies) [191,200]. Peripheral blood mononuclear cells (PBMCs) interact dynamically with astrocytes. Astrocytes secrete glycoproteins of the Wnt class (Wnt 1, 2b, 3, 5b, and 10b), involved in signal transduction in the CNS. Wnts reduce HIV replication in PBMCs, while IFNγ secreted by PBMCs increases the susceptibility of astrocytes to infection, leading to their further activation and adjustment of the Wnt profile by increasing Wnt 2b and 10b concentrations (in vitro study) [201].

#### 4.2.5. Telomere Shortening

Cells can enter a state of senescence, or an irreversible halt in the cell cycle, due to various factors, including epigenetic changes, telomere shortening or malfunction, exposure to mitogenic or oxidative stress, and the accumulation of DNA damage. Senescent cells often adopt a secretory profile that can provoke inflammatory responses in surrounding tissues. As organisms age, the accumulation of senescent cells increases, potentially leading to tissue inflammation, disruption of homeostasis, and the promotion of the aging process [202].

A longitudinal study by Leung JM et al. [203] found a significant reduction in telomere length in PLWH over an average period of two years or less, with a decrease of approximately 650 base pairs annually, although larger-scale studies are needed to validate these findings. A deficit of telomerase—the enzyme essential for lengthening and stabilizing chromosome ends—can lead to accelerated telomere shortening and activate the DNA damage response (DDR). Persistent DNA damage that remains unrepaired can result in programmed cell death (apoptosis) or drive cells into replicative senescence [148].

HIV-1 impairs telomerase activity, particularly in infected CD4+ cells, making blood cells more vulnerable to apoptosis and contributing to immune system dysfunction [204]. Furthermore, in vitro infection of human peripheral blood mononuclear cells (PBMCs) with HIV-1 shows a reduction in telomerase activity [205] within both nuclear and cytoplasmic compartments [204].

A study by Franzese O et al. indicated that in the context of HIV-1 infection, either the virus itself or its Tat-protein can disrupt telomerase activity, leading to a reduced expression of human telomerase reverse transcriptase (hTERT) in PBMC, with a particularly marked effect in the nuclei of CD4+ T cells [66]. The HIV-1 Tat-protein triggers pro-inflammatory responses in microglia, astrocytes, and neurons, stimulating the release of potent pro-inflammatory cytokines, including tumor necrosis factor-alpha (TNF-α) and interleukin-1 beta (IL-1β), which contribute to neuronal injury and cognitive decline (in vitro study) [206,207]. Moreover, a study by Hsiao CB et al. [23] highlighted the effects of HIV Tat on human microglial cells, noting a substantial decline in telomerase activity, telomere length, and mitochondrial function, along with elevated oxidative stress.

HIV infection is associated with an increased production of reactive oxygen species, DNA damage, the senescence-associated secretory phenotype, the metabolic reprogramming of infected cells, G1 cell cycle arrest, telomere shortening, and epigenetic modifications of DNA and histones [208]. Recent data have shown that oxidative stress accelerates telomere shortening in vitro [209]. Pollicita M. et al. [67] found that telomeres are a target in vitro for oxidative damage in PLWH and may act as critical indicators of cell apoptosis triggered by oxidative stress. Analysis of telomeres showed statistically significant shortening in HIV-exposed samples compared with controls in HIV-1-exposed human astrocytoma cells, aligning with the increased rate of apoptosis observed in these cells [67]. Reactive oxygen species contribute to single-strand breaks in telomeric DNA, causing the loss of distal telomeric fragments during replication [210,211]. Additionally, combined antiretroviral therapy (cART) resulted in a 74% reduction (*p* < 0.01) in telomerase gene expression compared with untreated controls, highlighting the complex interactions between HIV proteins and telomere shortening [23].

Telomere shortening may also accelerate as a side effect of nucleoside reverse transcriptase inhibitors used in antiretroviral therapy (ART) (ex vitro study) [212]. However, research suggests that accelerated telomere shortening is primarily caused by HIV-1 rather than ART exposure [213,214]. Shiau et al. [215] found that both HIV-1-positive and HIV-1-exposed negative children had shorter telomere lengths than children who were unexposed to HIV-1.

Interestingly, HTHU cells treated with cART did not show a notable decline in telomerase gene expression. Conversely, the HTHU/HIV microglial cells treated in vitro with 10 ng/mL and 100 ng/mL of HIV-Tat demonstrated a significant decrease in telomerase gene expression, with reductions of 59% (*p* < 0.05) and 62% (*p* < 0.05), respectively [23].

#### 4.2.6. Neurogenesis Impairments

Neurogenesis is a complex process involving the formation of new neurons and glial cells from neural stem cells (NSCs) and neural progenitor cells (NPCs) within specific brain regions, notably the subventricular zone (SVZ) and the subgranular zone (SGZ) of the hippocampus. Recent studies indicate that HIV-1 can infect NSCs and NPCs, either productively or non-productively [216]. According to Ferrell D et al., the CXCR4 (CD184) receptor—an α-chemokine receptor used by HIV to infect CD4+ T cells—may serve as an entry point for HIV into neural progenitor cells, as CD184 is expressed in new neurons from embryonic stages through adulthood, playing a key role in guiding neuronal development (in vitro study) [217].

The presence of HIV-1 proteins, along with immune and inflammatory responses associated with infection, can hinder the differentiation of NSCs into NPCs, reduce differentiation into neuronal lineages, and abnormally increase differentiation into astrocytic lineages [216].

Under normal conditions, microglia are highly concentrated in the SVZ and SGZ, where they significantly influence adult neurogenesis by secreting cytokines and chemokines, especially following brain injuries (in vitro study) [126]. Consequently, the inflammatory activation of microglia in PLWH plays a major role in neurogenesis impairment.

In vitro studies on human primary NSCs have shown that HIV-1 can not only directly infect these cells but also spread from infected NSCs to uninfected ones. Clinical studies have confirmed the presence of the HIV-1 proviral genome in both the SVZ and SGZ of pediatric AIDS patients, although larger-scale studies are needed to validate these findings [216,218,219].

Persistent HIV-1 infection in NSCs may disrupt essential functions, such as self-renewal, tripotential differentiation, and overall survival. Recent findings suggest that HIV-1 infection not only reduces differentiation into neuronal lineages but also impairs the morphological development of differentiated neurons [216].

Skowronska et al. revealed that methamphetamine can activate HIV replication within NPCs, a process mediated through the activation of the HIV long terminal repeat (LTR) via NFκB and SP1. This interaction may lead to abnormal NPC differentiation and impaired neurogenesis, creating latently infected neurons and glial cells. Such findings help explain the increased cognitive impairments seen in HIV-infected individuals who also use methamphetamine [220].

Early research using human primary NSCs and hippocampal slices indicates that HIV-1 coat proteins, or cerebrospinal fluid from PLWH, can suppress the differentiation of NSCs into NPCs by inducing a quiescent state in stem cells (ex vivo study) [221].

Additionally, the HIV-1 Tat protein has been shown to inhibit adult neurogenesis by reducing NSC proliferation and neuronal lineage differentiation without affecting NSC viability [221]. This decline in NSC proliferation is associated with lower cyclin D1 expression and decreased activation of the ERK1/2 signaling pathway (in vitro study) [222].

The HIV-1 coat protein gp120, depending on the viral strain, binds to CCR5 and/or CXCR4 receptors on neural progenitor cells, interfering with normal neural differentiation [223].

Integrated proviral DNA possibly remains within NSCs as they differentiate into neurons, astrocytes, or oligodendrocytes. Alternatively, the latent provirus might reactivate when NSCs differentiate into rapidly dividing NPCs, potentially allowing the infection to spread to nearby immune cells and astrocytes. This secondary infection could either re-establish the latent reservoir within NSCs or damage both the NSCs and their descendant cells [216].

In an in vitro study, Lawrance DM et al. [224] found that reactivation of latent infection in neural progenitors could also be triggered by inflammatory cytokines such as TNF-α. This raises concerns that brain progenitor cells may be stimulated to produce the virus at any stage throughout the prolonged course of the disease.

For a better overview, the HIV-related molecular mechanisms in accelerating brain aging and their pathophysiological pathways are summarized in Table 2.

**Figure 2:**
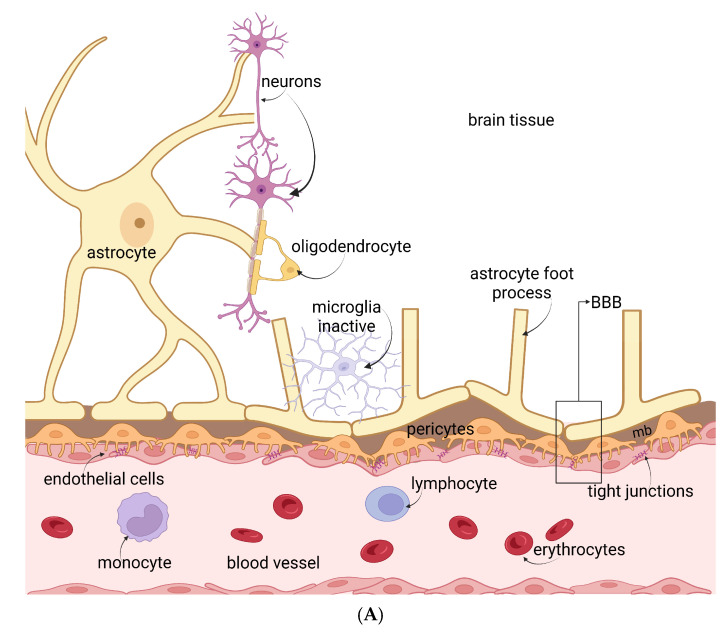

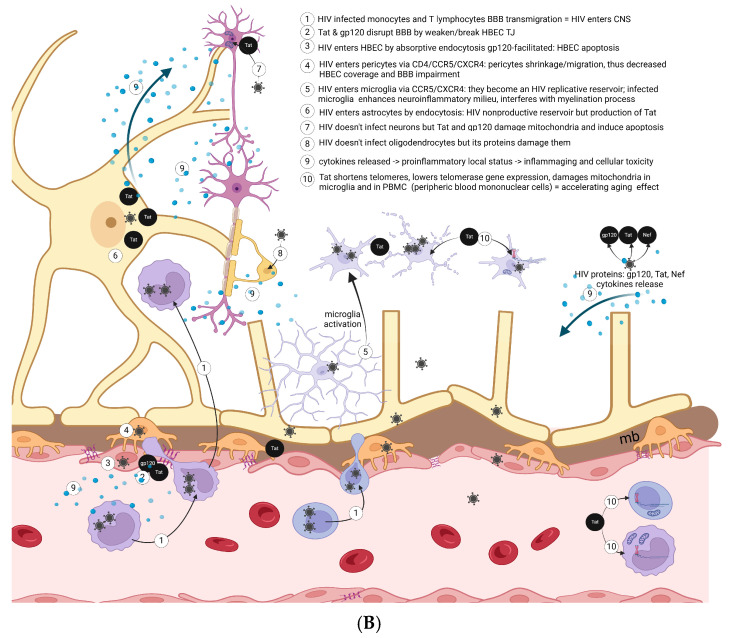
(**A**) Normal aspect of the blood-brain barrier and central nervous system; (**B**) Interactions of HIV with the blood-brain barrier and central nervous system cells. Created in BioRender. Moroti, R. (2024) BioRender.com/m90u929, 8 November 2024. Abbreviations: BBB—blood-brain barrier, CCR—cysteine chemokine receptor, CD—cluster of differentiation, CNS—central nervous system, CXCR—CXC chemokine receptor, HBEC—human brain endothelial cells, HIV—human immunodeficiency virus, PBMC—peripheral blood mononuclear cells, TJ—tight junctions.

The interactions of HIV with the blood-brain barrier and the central nervous system cells are represented in Figure 2A,B.

### 4.3. HIV Treatment and Its Impact on Brain Aging

Using combined ART in HIV infection has significantly extended the life expectancy of people living with HIV (PLWH) by over 20 years per individual, allowing for the observation and assessment of chronic complications related to both HIV and ART administration [20,23,225,226,227,228].

Despite the efficacy of ART in reducing the viral load and improving cognitive function, HAND is still persistent among patients [11]. Some questions arising in this context are as follows: Is it due to the inability of ART to suppress the HIV concentration in the brain, despite its suppressive action in the blood, because the latent HIV reservoirs in the brain could not be easily reached and properly depleted by ART [11,13,229]? Or is it because ART may cause some neurotoxic effects?

The research is vast and heterogeneous and addresses pathophysiological and clinical aspects.

Although numerous studies confirm the effectiveness of ART in enhancing the cognitive function in PLWH [230], a 2010 study on 144 HIV-positive asymptomatic, early-treated patients who interrupted ART after a median time of 4.5 years, in a controlled manner, showed significant improvements in mean neuropsychological scores over the following 96 weeks after the interruption, especially if they had an efavirenz (EFV) treatment-based regimen [231]. However, in this study, ART consisted of old nucleoside reverse transcriptase inhibitors (NRTIs): zidovudine, stavudine, and abacavir; first-generation non-nucleoside reverse transcriptase inhibitors (NNRTIs) and protease inhibitors (PIs) that are today on the second line of therapy (EFV, PI) or not in use (zidovudine, stavudine). The worst score was obtained in the EFV-containing regimen.

Another study comparing untreated HIV-positive patients or those on ART with age-matched or older non-demented HIV-negative individuals observed elevated levels of hyperphosphorylated Tau in the hippocampus of many HIV-infected subjects, compared with age-matched controls, with the greatest levels noted in patients on ART. However, these findings were unrelated to clinical cognitive impairment [232].

These abovementioned studies were among those earlier that raised concerns about the potential neurotoxicity of the treatment.

More recent studies, such as the one by Hsiao et al. (2021), demonstrated in vitro neurotoxicity of actual first-line antiretrovirals: immortalized transformed human microglia (HTHU) and HIV-infected HTHU (HTHU/HIV) treated with HIV Tat (different concentrations) or with ART (tenofovir, emtricitabine, and dolutegravir) had a significant decrease in telomere length, more pronounced in HTHU/HIV due to ART (a decrease of 77% in telomere length versus a reduction of 43% in HTHU), and a significant decrease in telomerase gene expression only in HTHU/HIV (and not in HTHU); a reduction of around 60% under HIV-Tat and 74% with ART. ART also resulted in a substantial decline in mitochondrial respiration in microglia by lowering the basal oxygen consumption rate, which led to impaired ATP production and increased ROS output. The ROS production is prevalent in “oxidative stress-induced aging”, causing oxidation, mitochondrial dysfunction, and early cell apoptosis, which supports the hypothesis that mitochondrial damage causes accelerated aging and neurocognitive decline in HIV patients. Additionally, alterations in microglial metabolism and cell integrity were observed [23].

Another relatively recent study (2019) showed that HIV-infected patients with extended ART exposure may suffer from age-related changes and accelerated aging due to decreased levels of neurotrophin-3 (NT-3) and high levels of matrix metalloproteinase-1 (MMP1). NT-3, an inflammatory marker that promotes neuron survival, differentiation, and neurogenesis, has been highly correlated to neurocognitive dysfunction in PLWH, while MMP1 has been associated with conditions of cellular senescence [24].

Highly active ART (HAART) involves a combination of three drugs (at least two) belonging to usually two different ART classes that efficiently inhibit HIV replication [11]. The main ART classes in use are reverse-transcriptase (RT) inhibitors—nucleos(t)ide RT inhibitors (NRTI) or non-nucleoside RT inhibitors (NNRTI), protease inhibitors (PI), and integrase strand transfer inhibitors (INSTI). The preferred combinations of starting ART today consist of two drugs (one NRTI plus one INSTI of the second generation) or three drugs (two NRTIs plus a third drug belonging to either the INSTI class or new-generation NNRTI; the regimens with the third drug belonging to PI are no longer among preferred HAART regimens) [228,233,234].

#### 4.3.1. Nucleoside Reverse Transcriptase Inhibitors (NRTIs)

Given concerns that ART may contribute to neurotoxicity and brain aging, studies have examined the effects of NRTIs on CNS cells, including neurons [21], microglia [23], and astrocytes [22]. These studies often found mitochondrial toxicity to be a key trait of aging and neurodegenerative diseases; other studies reported that NRTIs are not linked to brain mitochondrial DNA (mtDNA) depletion or increased mutations, key factors in aging among PLWH (in vitro study) [235]. This could be due to the dominant effects of HIV over ART or because NRTI-induced mitochondrial toxicity is dose-dependent, with CNS levels not being sufficient to cause significant mtDNA damage [235].

The first discovered and used NRTIs were zidovudine, stavudine, didanosine, and zalcitabine. They provided important mitochondrial toxicity (see further) and are no longer in use. The NRTIs contained in the current ART regimens are abacavir, lamivudine, emtricitabine, and tenofovir (disoproxil or alafenamide).

Schweinsburg et al. (2005) observed a significant connection between extended treatment with didanosine and/or stavudine, drugs associated with mitochondrial toxicity (in vitro study) [236], and decreased N-acetylaspartate (NAA) levels in the frontal WM, indicating compromised brain mitochondrial viability [27]. Prolonged use led to greater reductions in NAA, and those on multiple NRTIs (stavudine, didanosine, or abacavir) showed a tendency towards even lower NAA levels compared to those taking only one NRTI—didanosine or stavudine [25]. NAA is not only related to mitochondrial dysfunction, as it is synthesized in mitochondria in an ATP-dependent process, but it also represents an important protein stabilizer and biomarker for neuronal integrity [27,237]. Two potential mechanisms for NRTI-induced reduction in NAA may be competitive inhibition of mitochondrial replication and mitochondrial electron transport disruption [27].

The old combination of zidovudine plus lamivudine, in contrast with the new combination, tenofovir plus emtricitabine in ART regimens, was linked to the development of anemia. Zidovudine plus lamivudine (mostly due to the zidovudine component) are frequently associated with macrocytic anemia as they inhibit globin gene transcription, hemoglobin synthesis, and erythroid progenitor cell differentiation and proliferation; anemia’s impact on the brain varies with its severity and duration: Lowered cerebral oxygenation can result in impaired synaptic function and neuronal apoptosis, particularly in areas such as the hippocampus, basal ganglia, neocortex, and thalamus (in vitro study) [238].

Abacavir’s neurotoxicity was studied in human astrocytes; it was found to induce endoplasmic reticulum (ER) stress, an organelle responsible for protein synthesis and folding, lipid biosynthesis, and calcium storage. Specifically, abacavir upregulates ER stress markers such as binding immunoglobulin protein (BiP), C/EBP homologous protein (CHOP), and calnexin, considered “pro-survival proteins“ [22]. BiP facilitates the removal of misfolded proteins from the ER, while CHOP plays a pro-apoptotic role by triggering mitochondrial depolarization, leading to the release of cytochrome C and the activation of caspase 3 [235]. Under prolonged ER stress conditions, a disrupted balance between BiP and CHOP can shift to cellular apoptosis; additionally, abacavir induces astrocyte-elevated gene-1 (AEG-1) upregulation, which directly interacts with the calcium-binding chaperone calnexin [22]. This interaction increases intracellular calcium levels, leading to mitochondrial permeability transition pore opening, resulting in mitochondrial dysfunction and early apoptosis through altered mitochondrial membrane potential, compromised ATP synthesis, and elevated ROS production [22,239]. Increased calcium influx also triggers calcium-dependent exocytosis, leading to excessive glutamate release from astrocytes; the resulting elevated glutamate in synapses overstimulates neurons, causing neuronal damage [22].

Abacavir has been linked to an increased cardiovascular risk since 2008, when there was the first report by the D:A:D (the Data Collection on Adverse Events of Anti-HIV Drugs) about its association with acute myocardial infarction [240]. This could be extrapolated to an increased cerebrovascular risk too.

Winston et al. (2010) compared three ART regimens in a prospective study about neurocognitive improvement and neuronal recovery at 48 weeks after the introduction of tenofovir plus emtricitabine and either (1) efavirenz, (2) atazanavir/ritonavir, or (3) zidovudine plus abacavir. Greater improvements in neurocognitive function were observed for recipients of regimen no. 3, tenofovir plus emtricitabine plus zidovudine plus abacavir (meaning four NRTIs), but greater neuronal recovery (NAA/cr, N-Acetyl Aspartate/Creatine ratio) was observed for regimen no. 1, tenofovir plus emtricitabine plus efavirenz (two NRTIs plus one NNRTI, known for its neurotoxicity). The authors suggested that the improvement in NAA/cr in frontal WM observed with the regimen containing efavirenz is possibly due to the greater suppression of HIV effects in the CNS, probably because of better efavirenz CNS penetrability [241].

The neurotoxic effects of tenofovir, a first-line drug today, were demonstrated in SH-SY5Y cells (derived from human neuroblastoma and which can differentiate into neuronal cells). Tenofovir exposure reduces neuronal viability and promotes apoptosis, as indicated by elevated lactate dehydrogenase (LDH) levels, suggesting plasma membrane damage. Tenofovir increases intracellular ROS production, leading to enhanced oxidative stress, indicated by diminished antioxidant enzyme activity and increased malondialdehyde levels. Furthermore, tenofovir enhances the expression of caspase-3, a key biomarker of apoptosis, and induces nuclear condensation and fragmentation in the neuronal cells [21].

Tenofovir has been demonstrated to have the most significant effect on telomere length reduction and telomerase activity reduction in the peripheral blood mononuclear cells (PBMCs) among NRTIs (ex vivo studies) [23,24,242]. In addition, elevated expression of the TRF-1 (telomere repeat-binding factor 1) gene, which is responsible for preventing telomere shortening, was noticed in ART-treated patients [23]. Leukocyte telomere length (LTL) is regarded as a ‘marker of biological aging’, and studies have linked it to inflammation-induced aging or aging-related pathologies, neurocognitive impairment, brain structural lesions contributing to the development of Alzheimer’s disease/Alzheimer’s disease-related dementia, cerebral subcortical atrophy, and WM hyperintensities [24,25,26]. One possible mechanism behind subcortical atrophy and WM hyperintensities includes decreased LTL, followed by inflammation (involving ROS), which contributes to small vessel disease and demyelination, affecting subcortical structures due to their vulnerability to ischemia caused by the higher expression of pro-inflammatory enzymes in that region [243].

#### 4.3.2. Non-Nucleoside Reverse Transcriptase Inhibitors (NNRTIs)

In current use are efavirenz (EFV), rilpivirine, and doravirine. EFV, one of the most used HIV drugs worldwide, is today considered a component of alternative/secondary regimens. EFV is largely associated with CNS adverse reactions, reported in more than 50% of patients, often necessitating discontinuation of therapy [238,244].

Studies on human neuroblastoma SH-SY5Y and glioma U-251MG cells demonstrated that both cell lines experienced mitochondrial respiratory dysfunction and impaired ATP production due to EFV exposure, with the latter showing greater susceptibility [243]. Thus, neurons and especially glial cells exhibited a significant reduction in basal oxygen consumption rate, maximum respiratory capacity, spare respiratory capacity, and respiratory control ratio, parameters influenced by oxidative phosphorylation and, therefore, mitochondrial function [243]. Additionally, the mitochondrial dysfunction of SH-SY5Y cells led to mitochondrial depolarization and, ultimately, to mitochondrial autophagy (mitophagy), fragmentation, and perinuclear mitochondrial clustering (in vitro study) [245].

EFV reduces claudin-5 levels, primarily from the tight junctions (TJs) in the BBB, leading to increased permeability and exacerbating HIV-associated cerebrovascular pathology (in vitro study) [246].

ER stress inhibitors, such as 4μ8c and 4PBA, reestablished the protein levels of claudin-5 but could not restore the endothelial integrity; moreover, 4μ8c further impaired endothelial barrier function [246].

Like zidovudine, EFV seems to induce anemia by causing erythrocyte shrinkage, cell membrane scrambling with phosphatidylserine translocation to the cell surface, and eryptosis (erythrocyte apoptosis) (in vitro study) [247], resulting in lower brain oxygenation and neuronal impairment [243].

Compared with lopinavir-ritonavir (PI drugs), patients on EFV for an average of 17.9 months exhibited poorer performance in several neurocognitive domains, particularly in verbal fluency, information processing speed, working memory, and executive functioning [238]. In contrast, other studies could not confirm the chronic neurotoxicity of EFV. Neuropsychological symptoms were observed in the first week of administration; however, they resolved by week four [248], and neurocognitive performance remained stable over three years of EFV therapy, with a decline observed only at higher concentrations of EFV [249].

Even though the neurotoxicity is considered an NNRTIs class effect, rilpivirine and doravirine have far less neurotoxicity. Doravirine was chemically designed to have as few neurotoxic effects as possible, and the safety profile of the DRIVE-AHEAD Study (2021) demonstrated favorable CNS adverse events versus EFV [250].

#### 4.3.3. Protease Inhibitors (PIs)

To date, only boosted darunavir remains in the international guidelines. The booster agent could be an old PI—ritonavir or cobicistat, a drug without intrinsic antiretroviral effect.

Among PIs, indinavir, lopinavir, and darunavir have very good CNS penetration, and the boost with ritonavir enhances it more. Conversely, atazanavir (especially non-boosted atazanavir) has one of the most modest CNS penetrations. A high CNS concentration is indispensable for good CNS viral clearance but, on the other hand, could be related to neurotoxicity.

Amprenavir and lopinavir reduce the level of the glutamate transporter EAAT2 (excitatory amino acid transporter 2) in astrocytes, and lopinavir decreases the concentration of EAAT1, with no direct cytotoxic effects (in vitro study) [251]. EAATs represent the major transport mechanism used in the CNS for maintaining a low extracellular glutamate level, with EAAT1 being more abundant in the neocortex and cerebellum and EAAT2 being predominant in the forebrain [252]. Lopinavir reduces intracellular L-glutamate and extracellular glutamine while increasing intracellular GABA [228]. Furthermore, lopinavir exposure can activate the ionotropic and metabotropic receptors for glutamate from astrocytes, leading to calcium influx. All these effects suggest that PIs can lead to neurologic dysfunctions through their negative impact on glutamate and GABA levels [253]. Moreover, these medications also reduce the levels of proteins Ki67 and PCNA, which are responsible for cell proliferation and do not affect ASCT1 and BDNF in astrocytes.

Darunavir alters the tau protein in the putamen, reducing the formation and destabilizing the neuronal microtubule network, thereby altering cytoplasmic streaming [254].

Ritonavir also correlates with enhanced Iba1 (ionized calcium-binding adaptor molecule 1) microgliosis in the putamen, altering the actin cytoskeleton during cellular movement (ex vivo study) [255].

Human astrocytes treated with either ABC and lamivudine or ABC, lamivudine, and ritonavir showed a significantly increased level of the senescence marker p21, suggesting that these combinations could lead to the induction of a senescence program (in vitro study) [256]. These treatments also increased various pro-inflammatory molecules, such as IL-6, thus activating the transcription of NF-kB which is responsible for inducing senescence and stimulating the release of mitochondrial ROS, responsible for oxidative stress, leading to mitochondrial dysfunction and accelerating brain aging [257,258].

In a study analyzing the correlation between inflammation, autophagy, and the p-38 MAPK pathway on U87 cells, administration of lopinavir, ritonavir, darunavir, indinavir, and saquinavir resulted in higher BBB permeability and influenced various markers of autophagy (LC3-II/LC2-I ratio, p62) (in vitro study) [259]. Lopinavir and saquinavir decreased the ratio of LC3-II/LC3-I and increased the level of p62, whereas darunavir and indinavir did not influence the U87 cells regarding autophagy processes. Furthermore, lopinavir induced the secretion of multiple inflammatory cytokines in U87 cells, such as IL-6, M-CSF, MIPI1a, PDGF-AA, VEGF-A, IL-8, IFN-g, TNF-a, and TNF-b. A genetic analysis performed to evaluate the role of p38 MAPK in lopinavir’s action showed that the expression of four genes related to the p38 MAPK pathway was upregulated in the cells receiving lopinavir: nerve growth factor (NGF), DNA damage-inducible transcript 3 (DDIT3), growth arrest, DNA damage 45A (GADD45A), and fibroblast growth factor 2 (FGF2). Additionally, Western blotting showed that lopinavir stimulated phosphorylation of the p83 MAPK, thereby attenuating autophagy [259].

PIs also stimulate the unfolded protein response, possibly by proteasome inhibition, and increase APP cleavage and beta-amyloid production in neurons by up-regulating the expression of β-site amyloid precursor protein (BACE1) and BiP (a molecular chaperone from the lumen of the endoplasmic reticulum) [260,261].

Soontornniyomkij et al. reported that treatment with PIs yields a higher likelihood of developing mild cerebrovascular disease (CVSD) (OR 3.1) [262]; however, the mechanism remains unclear, but it is hypothesized that PIs may alter the cellular components of small cerebral vessels by stimulating the accumulation of farnesylated prelamin A in vascular endothelial cells, a marker of premature senescence, or by triggering metabolic disturbances (ex vivo study) [263].

#### 4.3.4. Integrase Strand Transfer Inhibitors (INSTIs)

INSTIs represent the newest ART drugs, with high efficacy, and the second-line INSTIs prove a remarkably high genetic barrier to virological resistance [264]. INSTIs are included in the preferred therapy/first-line agents in all international guidelines, but they could exert neurotoxic effects on the CNS, especially neuropsychiatric ones [233,234].

Having a good CNS penetration effectiveness score (CPE), raltegravir’s effects on the brain have been evaluated by many researchers; it demonstrates pro-inflammatory effects alone but anti-inflammatory effects in the presence of HIV on primary brain-derived macrophages (in vitro study) [265,266]. It had no impact on β-III tubulin, a marker of astrocytosis, and significantly decreased IL-8 (responsible for the chemotaxis of monocytes and neutrophils), while increasing IL-10 (an anti-inflammatory molecule), revealing a low neurotoxic profile for raltegravir [266].

Following this, the Swiss HIV Cohort Study demonstrated that discontinuation of the treatment with raltegravir occurred in less than 2% of the patients due to neuropsychiatric side effects [267].

There are many contradictory results regarding dolutegravir’s neuropsychiatric effect: some report a high frequency of psychiatric adverse reactions [268,269], while others reveal a low occurrence of those [267]. However, a recent meta-analysis, after analyzing only randomized controlled trials, showed that dolutegravir has a comparable neuropsychiatric risk to efavirenz [270]; the same study revealed that dolutegravir and rilpivirine increase the depression rate synergistically. Another study showed that therapy with dolutegravir leads to neuropsychiatric events, mostly insomnia, sleep disturbances, dizziness, and painful paresthesia, at a higher rate in older and female patients [271].

Dolutegravir’s effects may be amplified in patients with UDP-glucuronosyltransferase 1A1 (UGT1A1) gene polymorphism due to a reduction of UGT1A1, the main enzyme that metabolizes dolutegravir, leading to increased plasmatic concentration of dolutegravir and higher rates of neuropsychiatric side effects [272].

Bictegravir, another broadly used INSTI, induces mitochondrial dysfunction and disrupts mitochondrial iron transport in CNS-derived human microglia and SH-SY5Y cells. Both cell types exhibited a substantial reduction (20–30%) in mitochondrial respiratory function, including basal oxygen consumption rate, maximal respiration, spare respiratory capacity, ATP production, and proton leak. The reduced proton leak could be attributed to decreased expression of uncoupling proteins; mild mitochondrial uncoupling is essential for the adaptive mitochondrial response to stress linked to longevity, known as mitohormesis. Bictegravir-treated SH-SY5Y cells presented elevated Nox2 expression (a crucial superoxide-generating enzyme involved in cellular ROS production, neuronal damage, and apoptosis) and reduced mitochondrial Sod2 (superoxide dismutase, a superoxide scavenger enzyme) and Lrfn2 (leucine-rich repeat and fibronectin type-III domain-containing protein 2—enrolled in neurite growth and indicating synaptic integrity in the brain). Regarding iron homeostasis, which is essential for proper mitochondrial function, SH-SY5Y cells presented increased Tfr expression (transferrin receptor) along with reduced levels of Fth1 (the ferroxidase-containing subunit of ferritin) and mitoferrin (a mitochondrial inner membrane protein) [20].

Mitochondrial dysfunction and oxidative stress reduce the expression of mitochondrial iron-containing proteins, impacting the complexes responsible for electron transfer, ATP production, and the mitochondrial membrane potential [239]. Morphologically, mitochondria also appeared fragmented following bictegravir exposure [20].

CNS-derived human microglia and SH-SY5Y cells exposed to two regimens of antiretroviral drugs (Regimen1: abacavir, dolutegravir, and lamivudine; Regimen2: bictegravir, tenofovir, and emtricitabine) showed elevated levels of total and/or mitochondrial ROS [4]. Regimen2-treated SH-SY5Y cells presented an increased mtDNA copy number, presumed to be due to a compensatory response to mtDNA damage and mitochondrial dysfunction mediated by ROS; these changes did not occur in Regimen2-treated HIV+ microglia [20]. Withal, cytosolic ROS preceded a significant reduction in non-mitochondrial oxygen consumption rate in both microglia and neuronal-derived cells; both cell types displayed cytosolic and mitochondrial iron accumulation [20]. Chronic iron build-up leads to iron-mediated oxidative stress and mitochondrial damage, thus contributing to cellular senescence and pronounced aging in PLWH.

The neurotoxicity mechanisms of cART that may accelerate brain aging in PLWH are summarized in Table 3.

## 5. Molecular Markers Useful in Monitoring and Diagnosing Brain Involvement in PLWH

### 5.1. Neurofilament Light Chain (NFL)

NFL, an important structural component of myelinated axons, is a promising candidate for a marker of HIV-associated cognitive impairment [273]. The authors attribute a significant increase in cerebrospinal fluid (CSF) of NFL (CSF-NFL) to the presence of HIV, which triggers inflammation in the CNS, or to the degenerative lesions of the peripheral nervous system associated with HIV infection [274]. Although CNS-NFL is not specific to HIV-associated cognitive impairment, correlated with the clinical evidence, it can aid in identifying this condition, serving as a sensitive marker of neuro-axonal injury. For patients who refuse or cannot undergo a lumbar puncture for CSF assessment, plasma levels of NFL (plasmatic NFL) can also be measured, presenting a strong correlation with HIV-associated cognitive impairment [273,275].

Interestingly, untreated neuro-asymptomatic patients with low CD4+ T-cell counts, particularly those below 50 or within the range of 50 to 199 cells/microliter, also exhibited increased CSF-NFL, suggesting a subclinical active neural injury [273,275] that could lead to the development of HIV-associated dementia within 2 years, as a retrospective cohort study shows [276]. Similar patterns were observed for plasmatic NFL, making it a less invasive marker than CSF-NFL, thus reflecting neuro-axonal injury.

In patients on antiretroviral therapy (ART), plasmatic NFL levels decreased from a median of 22.7 pg/mL to 13.4 pg/mL 24 weeks after initiation, with corresponding improvements in neuropsychological test results improved accordingly [277], suggesting the impact of ART initiation on cognitive function. For the adult population undergoing ART, CSF-NFL levels were lower than those in untreated patients but higher in healthy controls, suggesting an aging effect of approximately 3.9 years. This may indicate either the impact of an initial injury or an ongoing infection and inflammation [275].

The applicability of NFL quantification in perinatally infected adolescents is noteworthy. A 2022 study by Julie van der Post et al. on perinatally HIV-infected adolescents shows that although the NFL levels continue to increase with age, there is no significant association between plasmatic NFL levels and the severity of HIV cognitive impairment (as determined by imaging and cognitive tests) in adolescents undergoing ART. This suggests that the pretreatment of early neuronal injury plays a more important role in this population than persistent neuroinflammation, which is at least partially suppressed by ART. Therefore, early diagnosis and treatment initiation may effectively prevent neurological complications in perinatally infected HIV individuals [278].

### 5.2. Neuroinflammation Markers

Despite the availability of effective ART, more than 50% of patients with HIV infection progress to HAND [279].

The main contributors to HAND development are believed to be microglia, as they are the principal cells infected by HIV in the CNS [280]. Although the pathogenesis of HAND remains unclear, activated microglia have been shown to induce neurotoxicity through their activation and inflammatory properties. They release pro-inflammatory markers, altered cytokines, chemokines, secondary messengers, and ROS, which activate signaling pathways, initiating neuroinflammation [279].

In response to neuroinflammation, several pro-inflammatory cytokines, including IL-1β, TNF-α, IL-6, granulocyte-macrophage colony-stimulating factor, and macrophage colony-stimulating factor, have been shown to increase in the CNS and/or the CSF of patients suffering from HAND [279].

#### 5.2.1. TNF-α

TNF-α plays a crucial role in HAND neuroinflammation and neurotoxicity, with elevated levels found in the brain tissues and CSF of HAND patients. TNF-α is increased by gp120 and Tat in macrophages, and TNF-α also promotes the release of other chemokines in the CNS, such as MCP-1, a chemoattractant for macrophages and monocytes [279]. Regarding neurotoxicity, TNF-α overstimulates glutamate receptors and increases glutamate release from microglia and astrocytes, resulting in higher glutamate levels in HIV-infected patients compared with uninfected individuals. Excess glutamate induces neuronal apoptosis and cell death [279,280]. TNF-α also promotes inflammation by inducing adhesion, chemoattraction, and activation of inflammatory cells. This is achieved by regulating the fractalkine ligand CX3CL1, which binds to its ligand, C-X3-C motif chemokine receptor 1 [279].

#### 5.2.2. Brain-Derived Neurotrophic Factor (BDNF)

BDNF is essential for learning and memory, with higher levels associated with a lower risk of developing HAND. BDNF plays important roles in proliferation, T-cell survival, and apoptosis by activating anti-apoptotic genes and reducing pro-apoptotic genes [281].

BDNF is obtained by the cleavage of a larger precursor protein, pre-proBDNF, into proBDNF, which is then cleaved into mature BDNF. The processing of proBDNF into mature BDNF has been shown to be reduced in patients with HIV. Increased TNF-α expression has also been shown to reduce BDNF expression in HIV [282], leading to dendritic injury and synaptic dysfunction present in HAND. Previous studies indicated that proBDNF levels in HAND subjects were higher than those in HIV-negative individuals and HIV-positive subjects without dementia [281]. However, low serum levels of BDNF in HAND may also be attributed to thrombocytopenia or increased TNF-a values, common conditions in PLWH. Genetic factors such as the rs6265 polymorphism (Val-Met amino substitution at codon 66) in the BDNF gene could also contribute to the lower BDNF levels observed in HIV-positive individuals [281].

#### 5.2.3. Alzheimer’s Disease Biomarkers

HAND has been associated with reduced levels of Alzheimer’s disease biomarkers (p-Tau181, Aβ42) and increased levels of interferon gamma-induced protein 10 (IP-10). However, high CSF levels of IP-10 have been associated with HIV infection in the absence of HAND. Elevated CSF levels of IL-8 have been found only in HIV-positive patients, not in those with HAND. Therefore, HAND may be characterized by reduced IL-8, increased IP-10, and decreased p-Tau181 [282].

#### 5.2.4. Proteome Analysis

Another method to demonstrate the presence of neuroinflammation in HIV-positive patients is proteome analysis of extracellular vesicles (EVs) extracted from the CSF. In a 2019 study [283], EVs were extracted from 20 patients with HIV, 10 of whom had cognitive impairment. An annotation was performed by gene ontology mapping, which showed proteins related to synapses, immune/inflammatory responses, stress responses, metabolic processes, mitochondrial functions, and the BBB. Patients with cognitive impairment exhibited a higher abundance of CSF EVs and proteins mapping to gene ontology terms for synapses, glial cells, inflammation, and stress responses. EVs have also been isolated by ultracentrifugation from cultured astrocytic U87 cells, detecting markers of astrocytes (GFAP, GLUL), inflammation (CRP), and stress responses (PRDX2, PARK7, HSP70) in EVs released by U87 cells under oxidative stress. These findings demonstrate that CSF EVs isolated from neurons and glial cells contain synaptic, immune/inflammation-related, and stress response proteins in HAND patients, although larger-scale studies are needed to validate these data [283].

### 5.3. Insulin Metabolism Parameter (sIR, s-IGF)

Research suggests that insulin metabolism plays a key role in the development and progression of HAND, preceding alterations of cognitive function [284]. Measurements indicated significantly higher soluble insulin receptor (sIR) levels in plasma, plasma exosomes, and CSF of HIV-infected women compared with controls [285,286]. In CSF, higher levels of sIR correlate with the presence and increased severity of HAND [287]. A positive correlation was also reported between exosomal sIR, exosomal ROS, and exosomal HIV Tat. TNFα significantly increases sIR levels in neurons treated with HIV Tat, with a dose-dependent maximal response at 100 nM concentration (larger-scale studies are needed to validate these findings) [287].

Soluble insulin-like growth factor-1 receptor (sIGF1-R) has been associated with higher cognitive dysfunction in HIV-seropositive women, similar to the association between full-length sIR and HAND [286,287].

In ex vivo studies of human microglia and neurons, insulin treatment reduced p24 levels. Human fetal neurons treated with insulin showed a decreased Vpr neurotoxicity, underlining the neuroprotective role of insulin in inhibiting HIV replication and pro-inflammatory gene expression. Insulin reduces the expression of IL-6 and CXCL10, with IL-6 seemingly correlating with HAND severity status (ex vivo study) [287,288].

### 5.4. Apolipoprotein E (ApoE)

ApoE, secreted by astrocytes, plays an important role in neuronal development and migration. The ApoE4 variant is linked to cognitive dysfunctions in HAND, atrophy of the corpus callosum, and speech impairment [289,290,291].

### 5.5. Opioid Intake

The intravenous use of opioids not only increases the risk of contracting HIV and other infectious diseases but also stimulates neuroinflammation and microgliosis [292,293] and elevates levels of hyperphosphorylated tau protein, associated with leukoencephalopathy and brain atrophy, although larger-scale studies are needed to validate these findings [294,295]. These processes lead to hyperalgesia, hyperkatifeia, and reduced volume in specific cerebral regions like the prefrontal cortex [295,296].

### 5.6. Neuroimaging in Aging HIV Patients

Newly developed noninvasive MRI techniques offer significant promise as potential biomarkers. Neuroimaging methods such as magnetic resonance spectroscopy (MRS), volumetric MRI, diffusion tensor imaging (DTI), and functional MRI (fMRI) provide valuable insights into the mechanisms underlying neuroHIV. These methods can aid in monitoring disease progression and evaluating the effectiveness of specific cART regimens [297].

Individuals with chronic HIV infection, even those receiving effective and stable cART, may experience progressive brain tissue loss. Tensor-based morphometry has been utilized to track annual changes in regional brain volumes in people living with HIV (PLWH), revealing substantial tissue loss, often localized to subcortical regions, even in asymptomatic patients [298].

In a cross-sectional study by Sanford et al. involving 119 treated and virologically suppressed HIV-positive individuals, HIV-positive participants exhibited lower cognitive performance, thinner cortical structures, and reduced subcortical volumes compared with HIV-negative controls. Additionally, a higher count of white matter hyperintensity lesions was linked to reduced cortical thickness, smaller subcortical volumes, and poorer cognitive function, regardless of HIV status [299].

Analyzing the correlation between cognitive impairment, abnormalities in MRI, and CSF markers of neurodegeneration in HIV-infected patients, Steinbrink et al. found a significant correlation between global brain atrophy, basal ganglia signal changes, CSF tau protein, and cognitive impairment in HIV-infected patients [300].

In examining structural and functional changes in PLWH, Liu et al. identified atrophy in the thalamus, occipital lobe, and hippocampal/parahippocampal regions. Functional connectivity (FC) was also altered within visual cortices, the thalamic-prefrontal circuit, and between the thalamus and somatosensory association cortex. Notably, in cognitively impaired PLWH, FC between the left thalamus and right dorsolateral prefrontal cortex differed significantly from that in cognitively normal PLWH, suggesting both brain atrophy and functional reorganization. These findings underscore the potential of MRI as a valuable tool for evaluating PLWH and enhancing the understanding of HIV-associated neurocognitive disorder pathogenesis [301].

The normal aging process is associated with a gradual increase in myoinositol and slight increases in choline and creatine, similar to changes observed in HIV-associated neural injury [302]. In a meta-analysis on brain metabolites in PLWH, Chelala et al. reported consistently lower total N-acetylaspartate/total creatine ratios, along with elevated total choline/total creatine and myoinositol/total creatine ratios linked to chronic HIV infection. They suggest that neurometabolite measurements can effectively detect the effects of chronic HIV and may be valuable for understanding the pathophysiology behind cognitive and sensorimotor decline associated with HIV infection [303]. Glutamate has also been reported as a marker of excitotoxicity in MRS evaluation in the basal ganglia and frontal white matter of treatment-naive patients, and it reduced after the initiation of cART [304,305].

HIV-positive individuals who were cART-naive exhibited a decline in N-acetylaspartate and total creatine in the basal ganglia that exceeded typical age-related reductions. They also showed a greater-than-expected increase in myoinositol (+12% rather than the usual 3% per decade) and choline (+10% instead of 2% per decade) in the frontal white matter, suggesting that HIV disease may contribute to accelerated aging [306]. Patients on cART also displayed signs of premature aging, evidenced by higher-than-normal myoinositol levels in the frontal white matter across all ages and reduced total N-acetylaspartate/total creatine ratios in the medial frontal cortex’s frontal gray matter [307]. Additionally, metabolite concentrations measured through MRS may serve as quantitative indicators of HIV’s impact on the brain, enabling assessment of cART efficacy in addressing HAND. Some researchers report that MRS parameters can show notable changes shortly after the initiation of antiretroviral therapy, indicating reduced neuroinflammation and neuronal loss. This is reflected in normalized or reduced levels of choline and myoinositol and normalized or near-normal levels of N-acetylaspartate compared with healthy controls, with improvements in metabolite concentrations observed as early as 3 to 6 months following the initiation of ART [302].

## 6. Conclusions

In people living with HIV, the process of brain aging is influenced by a combination of factors, including direct viral effects, chronic inflammation, neurotoxicity from antiretroviral therapy, and dysfunctions in the blood-brain barrier. These factors collectively accelerate the brain’s normal aging process, leading to an early decline in cognitive function, memory, and motor skills compared with the general population.

## Figures and Tables

**Table 1:** Factors involved in normal brain aging and HIV impact on normal brain aging.

Parameter	Mechanism Leading to Normal Brain Aging	HIV Impact on Normal Brain Aging Factors
Smoking	Long-term stimulation of cerebral nicotine receptors [37].	PLWH smokers develop greater neuronal damage, loss of myelin with brain atrophy, and poor cognitive performance [40].
Low physical activity	Diminished cerebral perfusion and synthesis of neurotrophic factors [38].	PLWH develop early musculoscheletal aging, especially sarcopenia and osteoporosis, which decrease physical activity [39].
Oxidative stress	Gene alterations, impaired mitochondrial activity, and increased ROS production [47]; Formation of advanced glycation end products (AGE), and activation of NF-kB with increased pro-oxidative and pro-inflammatory response [96,98,99,100,101,102,103].	Accelerated “oxidative stress-induced aging” with oxidation, mitochondrial dysfunction, early cell apoptosis, and, finally, neurocognitive decline [23]; gp120 increases oxidative stress and lipid peroxidation, with increased ROS and malondialdehyde and decreased intracellular glutathione, glutathione peroxidase, and superoxide dismutase activity [64], inducing apoptosis and cellular senescence [65]; HIV Tat induces significant decrease in telomerase activity [23,66], telomere length, and mitochondrial function, alongside an increase in oxidative stress in human microglial cells [23]; telomere structure represents a target for oxidative damage and a possible key sensor of cell apoptosis induced by oxidative stress in PLWH [67].
Slow metabolism	Accumulation of excessive catabolism products, such as beta and tau amyloid [50,51].	HIV Tat increases the production of beta-amyloid, inhibits its degradation and interactions with it [57]; HIV Tat promotes neurotoxicity by increasing phosphorylation of Tau protein [57].
Age-related DNA damage	Activation of NF-kB pathway and STING pathway [85,104,105]; microglia display SASP with increased inflammatory activity and elevated IFN signaling [85,107].	Deficit of telomerase may result in excessive telomere shortening and trigger DDR, with subsequent programmed cell death (apoptosis) or initiation of cells replicative senescence [148].
Oligodendrocytes dysfunction	Loss of myelin and WM atrophy [61,70,71].	HIV Tat interacts with NMDAR with subsequent increase in Ca^2+^ and CaMKIIβ, by which it induces oligodendrocytes immaturity; decrease in myelin-like membranes in mature oligodendrocytes or cell death [103].
Microglial dysfunction	Release of proinflammatory cytokines and free oxygen radicals with dysfunction of oligodendrocytes and BBB [61,65,112,113,114]; reduced clearance of myelin debris, and myelin sheath degeneration [75,115]; reduced recruitment and differentiation of OPC, decreasing the myelination process [85,93,94,95].	Microglial inflammatory activation in PLWH has an important role in neurogenesis impairment [126]; HIV-microglia infection increases the production of neurotoxic mediators, accelerating the normal loss-rate of myelination and loss of white matter volume [18].
Astrocyte dysfunction	Proinflammatory phenotype, releasing inflammatory cytokines and ROS, which are toxic to oligodendrocyte lineage cells [110,111]; enhances the recruitment and activation of peripheral immune cells in the CNS.	Astrocytes are regarded as reservoirs for HIV within the brain, which produce proteins as Tat with role in neuronal toxicity and synaptodendritic injury [129,130]; astrocyte infection increases the neuroinflammation process and occurrence of HAND [19].
Bioenergetic deficit	Decreased expression of GLUT [120,122]; deterioration of respiratory enzymes with mitochondrial dysfunction; decreased glycolytic capacity, increased oxidative stress, and neuronal apoptosis [120,123]; decreased aerobic glycolysis [120,124]; decreased ATP production [63,125,126]; declining NAD levels with glucose hypometabolism and impaired sirtuin function led to genomic instability, lowered GABAergic tonus, and increased energy demand [122,123].	HIV infection is associated with increased glucose metabolism; increased GLUT1 expression by proinflammatory monocytes is a potential marker of inflammation in HIV-infected subjects [138]; Increased oxidative stress, decreased oxidative phosphorylation, gluconeogenesis, ATP production, and beta-oxidation, abnormal cell homeostasis, upregulation of mitochondrial DNA mutations, and cell apoptosis [139]; Progressive mitochondrial damage induced by HIV-infection and antiretroviral treatment accelerates aging, senescence, and cell dysfunction [139].
CSVD	Progressive decrease in vascular lumen, with reduction of cerebral blood flow, chronic cerebral hypoperfusion, microbleeds, and microinfarcts [127].	HIV Tat accelerates the production of beta-amyloid and phosphorylation of Tau protein; complex interactions of HIV Tat with their structures that promote the production of neurofibrillary tangles, a hallmark of Alzheimer’s disease [57].
BBB dysfunction	Decreased oligodendrocyte precursor cell maturation, impairing myelination and myelin repair [133]; reduced expression of transporters such as GLUT and Pgp, leading to the impairment of both glucose influx and neurotoxic molecule efflux [134]; relocation of the aquaporin-4 molecules to the opposite side of the astrocyte end-foot, disrupting the normal interstitial fluid flow [133]; neuroinflammation and impaired nutrient transport, with further vasodilatation and ultimately cognitive decline [133,134].	Disruption of structure and function of BBB by reducing the pericytes coverage, altering cellular signaling and their interaction with the endothelial cells [15]; decreased BBB integrity with reduction of vital nutrients entering the brain; reduction of the blockage of pathogens, inflammatory cells, and other toxic agents, leading to early brain aging [16,17].

Abbreviations: AGEs—advanced glycation end products, ATP—adenosine triphosphate, BBB—blood-brain barrier, CaMKIIβ—Ca^2+^/calmodulin-dependent protein kinase II, CNS—central nervous system, CSVD—cerebral small vessel disease, DDR—DNA damage response, HIV—Human Immunodeficiency Virus, IFN—interferon, GABA—gamma-aminobutyric acid, GLUT—glucose transporter, NAD—nicotinamide adenine dinucleotide, NMDAR—N-methyl-D-aspartate receptor, NF-kB—transcription nuclear factor kappa-B, OPC—oligodendrocyte progenitor cells, PLWH—people living with HIV, ROS—reactive oxygen species, SASP—senescence-associated secretory phenotype, STING—cyclic GMP-AMP synthase-stimulator of interferon genes, TJP—tight junction protein, WM—white matter.

**Table 2:** HIV-related molecular mechanisms in accelerating brain aging.

Parameter	HIV-Related Molecular Mechanisms
Tat protein	Enhances HIV polymerase processivity, increasing transcription and boosting viral mRNA production by over 100-fold [158]; increases PDGF-BB expression in pericytes by activating the MAPK pathways, promoting pericyte migration [157]; creates a more pro-inflammatory brain environment by altering gene regulations; weakens (by inhibiting occludin mRNA expression via the RhoA/ROCK signaling pathway) and even breaks HBEC TJ integrity (by promoting occludin cleavage through MMP-9), increasing monocyte and T lymphocyte migration across the BBB [161,162,164]; induces tyrosine phosphorylation of the NMDAR subunit 2A, modifying protein-protein interactions and alterations in mitochondrial trafficking [197]; can induce apoptosis in human neurons, by the binding LRP [197]; shortens telomere length and telomerase gene expression in microglia, resulting in senescence [23].
gp120 protein	Mediates HIV entrance into endothelial cells through adsorptive endocytosis [158]; induces programmed death of HBEC in a caspase-3-mediated manner [170,171]; exhibits cytotoxic effects in the presence of IFN-γ via the p83 MAPK cellular pathway [172]; increases the oxidative stress-inducing apoptosis and cellular senescence [65]; disrupts BBB by activating local metalloproteinases [172]; elevates Ca^2+^ flux and activates PKC in HBEC, resulting in decreased BBB tightness and increased monocytic passage across it [174]; increases secretion of IL-6 and IL-8 in HBEC via the STAT pathway, promoting monocytic adhesion and migration across the BBB [175]; disrupts calcium balance, induces the pro-apoptotic transcription factor p53, activates oxidative stress, and alters mitochondrial function in neurons [198]; triggers the perinuclear accumulation and aggregation of damaged mitochondria, followed by mitochondrial fission in neurons [199]; impairs mitochondrial function by reducing mitochondrial membrane potential [199].
Nef protein	Increases IL-6 and IL-8, linked to higher endothelial layer permeability [178,179]; reduces MHC II and MHC I expression and increases the expression of immature MHC II complexes, helping the virus evade the immune response and further disrupting the BBB [180].
Pericyte infection	Decreased pericyte coverage of HBEC reduced Ang-1 production with less pericyte coverage and instability of the BBB [15,152]; increased production of growth factors and the pro-inflammatory cytokine IL-6, inducing neuroinflammation and decreasing BBB integrity [153,154]; altered pericyte gene expression modifies membrane plasticity [15].
Monocytes transmigration	Main mechanism that contributes to HIV infiltration into the CNS [181]; CCL2-chemokine and junctional proteins JAM-A and ALCAM play a key role in monocyte migration across the BBB in the acute phase of infection [184,185]; persistent eATP levels promote selective transcellular transmigration of infected monocytes through the BBB in the chronic phase of HIV infection [183].
Microglia infection	gp120 binds to CCR5 and CXCR4, entering the cells [194]; gp120 induces NLRP3-dependent pyroptosis and IL-1β production in microglia [18]; increased production of neurotoxic mediators accelerates the normal loss rate of myelination and loss of WM volume [18].
Astrocyte infection	Occurs mainly through cell-to-cell contact and endocytosis [129,191]; infected astrocytes produce non-structural proteins such as Tat [194]; Tat protein upregulates Cx43 expression and maintains and facilitates Tat transfer to uninfected cells through gap junctions [196]; alters gene regulation, increasing the neuroinflammation process and occurrence of HAND [19].
Macrophage infection	CD16+CD14+ type shows an improved capacity of transmigration compared with the low CD14 type [189]; diapedesis across the BBB is facilitated by junctional proteins such as JAM-A, ALCAM, PECAM-1, and CD99 [189]; PBMCs engage in a dynamic interaction with astrocytes, increasing the secretion of Wnt 1, 2b, 3, 5b, and 10b; IFNγ secretion by PBMCs increases the susceptibility of astrocytes to infection [201].
Telomere shortening	HIV-1 causes accelerated telomere shortening [66,215]; HIV leads to the generation of reactive oxygen species, DNA damage, the senescence-associated secretory phenotype, metabolic reprogramming of infected cells, G1 cell cycle arrest, telomere shortening, and epigenetic modifications of DNA and histones [208]; telomere structure represents an in vitro target for oxidative damage in PLWH [67]; HIV Tat decreases telomerase activity [23,66], telomere length, and mitochondrial function, alongside increased oxidative stress [23]; oxidative stress represents a cause for the acceleration of human telomere shortening in vitro [209]; cART decreases telomerase gene expression [23,148].
Neurogenesis impairments	HIV-1 can both productively and non-productively infect NSCs and NPCs [216]; CXCR4 (CD184) represents a possible entry point for HIV into NPCs [217]; HIV-1 proteins, along with associated immune and inflammatory responses, limit the differentiation of NSCs into NPCs [216]; microglial inflammatory activation in PLWH plays an important role in neurogenesis impairment [126]; HIV-1 Tat protein reduces NSC proliferation by decreasing cyclin D1 expression and ERK1/2 signaling pathway activation [221,222]; HIV-1 Tat protein reduces NSCs’ neuronal lineage differentiation [221]; the integrated proviral DNA may remain as NSCs differentiate into neurons, astrocytes, or oligodendrocytes [216]; reactivation of a latent infection of the neural precursors may be triggered by NSC differentiation [216] or by inflammatory cytokines [224].

Abbreviations: ALCAM—activated leukocyte cell adhesion molecule, Ang-1—angiotensin 1; ART—antiretroviral therapy, ATP—adenosine triphosphate, BBB—blood-brain barrier, CCL—chemokine (C-C motif) ligand 2, CCR5—C-C chemokine receptor type 5, CD4—cluster of differentiation 4, CNS—central nervous system, CXCR4—C-X-C chemokine receptor type 4, Cx43—connexin43, DNA—deoxyribonucleic acid, eATP—extracellular ATP; ERK—extracellular signal-regulated kinase, HAND—HIV-Associated Neurocognitive Disorder, HBEC—Human brain endothelial cells, HIV—human immunodeficiency virus, IFN—interferon, IL—interleukin, JAM-A—junctional adhesion molecule A, LRP—Lipoprotein receptor-related proteins, MAPK—mitogen-activated protein kinase, MHC—major histocompatibility complex, MMP-9—Matrix metalloproteinase-9, mRNA—messenger ribonucleic acid, NLRP3—NLR family pyrin domain containing 3, NMDAR—N-methyl-D-aspartate receptor, NPC—neural progenitor cell, NSC—neural stem cell, PBMC—peripheral blood mononuclear cell, PDGF-BB—platelet-derived growth factor BB, PECAM—platelet endothelial cell adhesion molecule, PKC—protein kinase C, RhoA—Ras homolog family member A, ROCK—Rho kinase, STAT—signal transducer and activator of transcription, ZO-1—Zonula Occludens-1.

**Table 3:** Neurotoxicity of antiretroviral drugs to the CNS.

Class of ART	Drugs	Neuropsychological/ Neuropsychiatric Adverse Reactions	Neurotoxicity Mechanism
NRTIs	Zidovudine * Didanosine * Stavudine * Zalcitabine * Abacavir **Lamivudine** **Emtricitabine** **Tenofovir**	Peripheral neuropathy; distal symmetric polyneuropathy; paraesthesia, confusion [233].	Mitochondrial toxicity in neurons, microglia, and astrocytes [20,21,22]; ER stress and calcium influx in astrocytes, followed by mitochondrial dysfunction and glutamate release in neuronal synapses, leading to neuronal apoptosis [235]; decreased NAA levels in the frontal WM [27]; telomere shortening and lowered telomerase expression in microglia and leukocytes (linked to inflammation-induced aging pathologies, neurocognitive impairment, brain structural lesions contributing to the development of Alzheimer’s disease/Alzheimer’s disease-related dementia, cerebral subcortical atrophy, and WM hyperintensities) [23,24,25,26]; macrocytic anemia, leading to lowered cerebral oxygenation, impaired synaptic function, and neuronal apoptosis, particularly in areas like the hippocampus, basal ganglia, neocortex, and thalamus [243].
NNRTIs	Nevirapine * Delavirdine * Efavirenz **Rilpivirine** Etravirine * Doravirine	Poor performance in verbal fluency, information processing speed, working memory, and executive functioning; mood alterations, insomnia, disturbing dreams, dizziness, headaches, suicidality, psychosis, mania; late-onset efavirenz neurotoxicity syndrome (LENS) presenting with ataxia and/or encephalopathy [233,238,246].	Mitochondrial toxicity in glial cells and neurons [238,246]; BBB integrity is impaired by lowering claudin-5 levels, especially in the TJ of BBB [247]; anemia, leading to low brain oxygenation, impaired synaptic function, and neuronal apoptosis, particularly in the hippocampus, basal ganglia, neocortex, and thalamus [243,248].
PIs	Saquinavir * Indinavir * Ritonavir ** Nelfinavir * Lopinavir * Atazanavir * Darunavir	Mood alterations, insomnia, dizziness [233].	Alter the glutamate transporter of the astrocytes [251]; reduce the intracellular L-glutamate and extracellular glutamine while increasing intracellular GABA [251]; increase proteins Ki67 and PCNA [251]; phosphorylate the tau protein [232]; increase pro-inflammatory molecules such as IL-6, M-CSF, MIPI1a, PDGF-AA, VEGF-A, IL-8, IFN-g, TNF-a, and TNF-b [259]; increase the transcription factor p56 mitochondrial ROS, mitochondrial proton leak, anaerobic glycolysis rate and extracellular acidification [256]; reduce the autophagy in U87 cells by phosphorylating the p83 MAPK [260]; stimulate the unfolded protein response and the production of BACE1 and BiP [260,261]; increase the likelihood of developing cerebrovascular disease (CSVD) [260,262].
INSTIs	**Raltegravir** Elvitegravir * Dolutegravir **Bictegravir** **Cabotegravir**	Neuropsychiatric side-effects (insomnia, anxiety, depression, headache, sleep disturbances, impaired thinking, low concentration ability, paraesthesia, and suicidality) [271]	Mitochondrial dysfunction in microglia and neurons [23]; dizziness, headache, insomnia, restlessness, and anxiety [262].

* out of use, to date; ** ritonavir is exclusively used to boost darunavir N.B.: booster agents in use are ritonavir or cobicistat; Abbreviations: BACE1—beta-site amyloid precursor protein cleaving enzyme; BiP—binding immunoglobulin protein; CSVD—cerebral small vessel disease; IL—interleukin, IFN—interferon, Ki67—marker of proliferation Kiel 67, MAPK—mitogen-activated protein kinase, M-CSF—macrophage colony-stimulating factor, MIPI1a—macrophage inflammatory protein-1 alpha, NAA—N-acetylaspartate; PCNA—proliferating cell nuclear antigen, PDGF-AA—platelet-derived growth factor AA, TNF—tumor necrosis factor, VEGF-A—vascular endothelial growth factor A.

## Data Availability

The data presented in this article are available upon request from the corresponding author.
